# Towards Stem Cell Therapy for Critical-Sized Segmental Bone Defects: Current Trends and Challenges on the Path to Clinical Translation

**DOI:** 10.3390/jfb15060145

**Published:** 2024-05-27

**Authors:** Jolene Quek, Catarina Vizetto-Duarte, Swee Hin Teoh, Yen Choo

**Affiliations:** 1 Developmental Biology and Regenerative Medicine Programme, Lee Kong Chian School of Medicine, Nanyang Technological University, Singapore 308232, Singapore; quek0226@e.ntu.edu.sg (J.Q.); catarina@ntu.edu.sg (C.V.-D.); 2 Centre for Advanced Medical Engineering, College of Materials Science and Engineering, Hunan University, Changsha 410012, China

**Keywords:** bone tissue engineering, mesenchymal stem cells, critical-sized bone defects, clinical translation, microenvironment, vascularisation, regulatory framework

## Abstract

The management and reconstruction of critical-sized segmental bone defects remain a major clinical challenge for orthopaedic clinicians and surgeons. In particular, regenerative medicine approaches that involve incorporating stem cells within tissue engineering scaffolds have great promise for fracture management. This narrative review focuses on the primary components of bone tissue engineering—stem cells, scaffolds, the microenvironment, and vascularisation—addressing current advances and translational and regulatory challenges in the current landscape of stem cell therapy for critical-sized bone defects. To comprehensively explore this research area and offer insights for future treatment options in orthopaedic surgery, we have examined the latest developments and advancements in bone tissue engineering, focusing on those of clinical relevance in recent years. Finally, we present a forward-looking perspective on using stem cells in bone tissue engineering for critical-sized segmental bone defects.

## 1. Introduction

Bone inherently possesses excellent self-healing ability. However, when the segmental defect is of a critical size, its capacity to naturally bridge defects is impaired, requiring surgical intervention [1]. Critical-sized segmental defects cannot heal spontaneously without surgical intervention [2] and in adults typically exceed two times the diameter of the bone or involve > 50% circumferential bone loss [3,4,5,6]. The reconstruction of large segmental bone defects, arising from congenital anomalies, trauma, infection, and tissue resection due to tumours, remains a major challenge for clinicians and surgeons [7,8].

**Figure 1:**
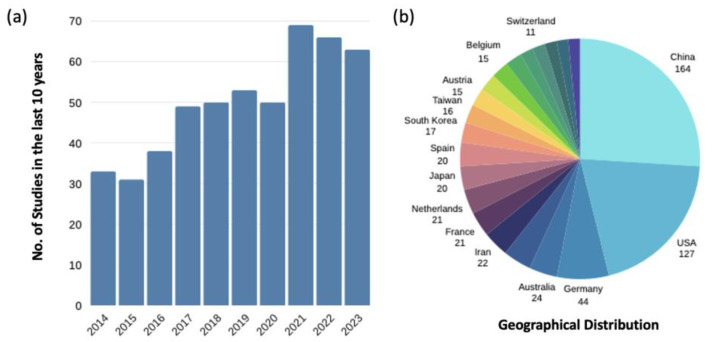
(**a**) Number of studies published in the last 10 years and (**b**) their geographical distribution.

Over two million bone graft surgeries are performed annually [9,10], making bone the most highly transplanted tissue after blood [11]. A literature search was conducted on the Web of Science using the terms ‘bone tissue engineering’, ‘clinical’, and ‘critical-sized bone defects’ to identify studies published within the last 10 years (Figure 1a) and their geographical distribution. There has been a steadily increasing number of studies and advancements in the reconstruction of critical-sized bone defects over the years (Figure 1), demonstrating the emergence of clinical-focused studies in bone tissue engineering (BTE). The top three countries leading in this field are China, the USA, and Germany. 

However, a gap persists between research-based knowledge and clinical practice, with research findings not readily translating into clinical application, leading to delays in the clinical adoption of new technologies [12]. The demand for bone grafts and substitutes is also on the rise, with the global market projected to grow at a compound annual growth rate of 5.8% from 2021 to 2028. By 2028, it is expected to reach a value of USD 4.3 billion [13]. Therefore, there is a pressing necessity to bridge the gap between research and clinical practice by incorporating clinical and regulatory requirements to enable more rapid clinical translation. This review focuses on the components of BTE, providing a comprehensive overview of the current clinical landscape in BTE and its limitations. Additionally, it offers future perspectives regarding the potential of stem cell therapy in critical-sized bone defects. 

## 2. Bone Tissue Engineering: Combining Stem Cells, Scaffolds, the Microenvironment, and Vascularisation 

Among the different techniques for treating bone defects, regenerative medicine approaches that incorporate stem cells in tissue engineering offer a promising alternative for fracture management, potentially eliminating the need for bone donors [14,15]. BTE operates at the intersection of bioengineering, biology, material science, and regenerative medicine, aiming to construct a suitable biological substitute to induce functional bone regeneration. Tissue engineering therapeutics typically comprise several components, including (1) stem cells, (2) biocompatible scaffolds, (3) a microenvironment incorporating signals for cell adherence and differentiation, and (4) sufficient vascularisation for nutrient and oxygen supply [16]. Various approaches for bone tissue engineering are explored, including considerations regarding the source of cells, the selection of a scaffold, and growth factors required for tissue formation.

### 2.1. Selection of Stem Cells

Studies on the incorporation of stem cells at the defect site following bone injury, particularly for critical-sized injuries, have increased in the past decade [17,18,19,20]. This trend is attributed to the multilineage potential and anti-inflammatory properties of stem cells [21], along with the improved bone regeneration observed in cellularised scaffold constructs [22,23]. Stem cell delivery is particularly attractive, especially for patients with compromised endogenous osteoprogenitor cells, as these cells can expand and differentiate into various cell types, given the appropriate stimuli [17,24]. Dupont et al. [17] showed that scaffolds seeded with human mesenchymal stem cells (hMSCs) and human amniotic fluid stem cells (hAFS) had more than two times the bone volume and torsional strength of an acellular scaffold, significantly enhancing bone regeneration in critical-sized segmental bone defects. 

Other studies have confirmed that MSC-seeded scaffolds show significantly higher bone formation compared to acellularised scaffolds [17,25,26], and MSC-mediated bone healing has been of longstanding interest in regenerative medicine. On the other hand, endogenous MSCs derived from the defect site in an inflammatory environment exhibit inferior bone regenerative potential [27]. A study by Caetano et al. [25] reported that polycaprolactone (PCL) scaffolds seeded with xenogeneic human adipose-derived stem cells (hASCs) exhibited 15% more bone formation than scaffolds without cells and scaffolds with differentiated ASCs (osteoblasts) in a rat bone defect model. The authors postulated that human ASCs released beneficial growth factors for bone regeneration, resulting in higher bone formation in the rat model. In other studies, xenotransplanted bone marrow-derived mesenchymal stem cell (BM-MSC)-seeded scaffolds were rejected after one week of implantation [28]; however, xenotransplanted ASC-seeded scaffolds were able to avoid immunosurveillance and aid the bone-healing process. ASCs express less human leukocyte antigen (HLA-ABC) than BM-MSCs, suggesting that ASCs could be a better alternative for transplantation [25]. Comparing BM-MSCs and ASCs, BM-MSCs have been reported to exhibit changes in proliferation and the senescence profile with increasing age, whereas ASCs do not display these unfavourable age-dependent effects, having relatively lower expression levels of the senescence markers SA-gal and p21 compared to BM-MSCs [29]. 

Another attractive cell source for regenerative medicine and tissue engineering is dental-derived stem cells (DSCs) due to their accessibility compared to that of bone marrow or adipose-derived MSCs, low immunogenicity, robust growth capacity, and multilineage potential [30]. This category includes dental pulp-derived stem cells (DPSCs), dental follicle-derived MSCs (DFSCs), stem cells derived from human exfoliated deciduous teeth (SHED), and periapical cysts MSCs (PCy-MSCs) [31]. DPSCs, first identified in 2000, possess multilineage potential and have demonstrated remarkable capabilities in nerve and bone regeneration. They are reported to exhibit superior differentiation potential into bone tissue compared to BM-MSCs in vitro [32]. SHED, identified in 2003, are derived from the pulp of deciduous teeth, which are typically discarded tissues. This source is highly accessible and promising, with SHED exhibiting higher proliferation rates and differentiation potential compared to DPSCs and BM-MSCs [31,33]. The most recent addition to dental-derived stem cells is periapical cyst-derived MSCs (hPCy-MSCs), discovered in 2013. These cells originate from periapical cysts, a pathological tissue. Clinical observations revealed the formation of new bone in the periosteum area following the surgical removal of periapical cysts, suggesting the involvement of stem cells in the regenerative process [34]. In comparing the osteogenic potential of hPCy-MSCs and DPSCs, hPCy-MSCs show a preference for osteogenesis, while DPSCs tend towards dentinogenesis. qRT-PCR analysis has shown higher expression levels of dentin sialophosphoprotein (DSPP) and DMP-1 genes in DPSCs compared to hPCy-MSCs. Conversely, hPCy-MSCs exhibit higher expression levels of osteonectin (ON), bone sialoprotein (BSP), and runt-related transcription factor 2 (RUNX2) compared to DPSCs [35]. Currently, dental pulp is the most commonly used cell source in clinical trials [36,37,38,39]. Although DSCs hold promise for bone tissue engineering applications, their clinical use, particularly with dental pulp, is still in its early stages [40]. Understanding the mechanisms underlying the therapeutic effects of these cells remains a significant challenge to be overcome before DSCs can be applied in human clinical settings [41].

Comparing DPSCs and ASCs, two of the most well-characterised cells for bone regeneration, reveals several advantages. Both types of cells have few ethical constraints, are easily accessible, and can be obtained through minimally invasive procedures compared to BM-MSCs. Additionally, they possess a high proliferation rate and multilineage potential. In vitro studies have shown that DPSCs exhibit a higher proliferation rate than ASCs, while ASCs demonstrate greater osteogenic differentiation potential than DPSCs. Moreover, in vivo studies in a rat model with mandibular defects have indicated faster and more substantial bone regeneration, as early as one week in the ASCs group, compared to DPSCs. This suggests that ASCs may hold greater utility than DPSCs for bone regeneration purposes [42].

#### 2.1.1. Heterogeneity of MSCs

MSC heterogeneity is evident in cell surface markers and gene expression profiles. The diversity among MSCs is attributed to factors such as the anatomical niche of origin and donor-specific variables including health and age, posing challenges to the clinical translation of these cells [43,44]. Importantly, at present, there is no single surface marker that universally defines MSCs [43,45]. 

The source of MSCs significantly influences their characteristics. ASCs, for example, demonstrate a superior proliferative capacity compared to BM-MSCs. ASCs also exhibit a higher potential for angiogenesis and vasculogenesis compared to BM-MSCs [44]. Umbilical cord-derived mesenchymal stem cells (UC-MSCs) exhibit an even higher proliferation rate than both ASCs and BM-MSCs. Different sources display lineage preferences in osteochondral development, with ASCs and BM-MSCs favouring osteogenic differentiation and UC-MSCs favouring chondrogenic differentiation. 

Within the same anatomical niche, variations in MSC characteristics are observable. ASCs obtained from subcutaneous adipose tissue may have higher osteogenic potential compared to those from deep-layer adipose depots [46]. The mode of sample collection also influences MSC properties, as demonstrated by differences in the proliferation rate, senescence resistance, and growth factor expression. ASCs obtained via power-assisted liposuction show a better proliferation rate and higher resistance to senescence than laser-assisted liposuction and surgical biopsy. Additionally, it has been reported that the microaspiration of adipose tissues with micro-cannulas results in a superior yield and viability and a higher expression of crucial growth factors, such as insulin-like growth factor (IGF) and platelet-derived growth factor (PDGF), than conventional liposuction [44]. This inherent heterogeneity poses challenges in deriving a homogenous and well-defined MSC population suitable for clinical applications. Addressing this challenge remains an ongoing need in the field [43]. Innovative ASC isolation and liposuction technologies could improve standardisation in a facile, reproducible manner [47].

To overcome the heterogenicity of stem cells, cell selection using unique cell-specific surface markers to selectively isolate and expand a specific cell type can be employed. In addition to commonly used MSC markers (CD73, CD90, CD105), additional characterisation can be implemented to specifically select a group of highly pure and homogenous cells displaying similar characteristics—for example, the selection of human MSCs using specific markers, such as CD271 or CD146. However, although CD271 has proven to be a suitable marker for BM-MSCs and ASCs, it is not suitable for the isolation of MSCS from other tissue sources, such as umbilical cord blood or Wharton’s jelly [48,49]. CD271 has also been described as the most specific marker for selectively isolating and expanding multipotent MSCs with immunosuppressive and lymphohematopoietic engraftment-promoting properties [48]. Colosimo et al. [50] demonstrated that CD271-selected rabbit BM-MSCs (rBM-MSCs) had better adhesion, proliferation, and osteogenic differentiation when cultured on a multiphase poly(ε-caprolactone)/thermoplastic zein-hydroxyapatite scaffold compared to non-selected rBM-MSCs. CD271-selected subpopulations of ASCs improved adipocyte, osteocyte, and neuronal cell differentiation compared to CD271-negative ASCs [51]. 

#### 2.1.2. Fetal Bovine Serum, Human Alternatives, and Serum-Free Media

The presence of xenogeneic components in the cell-manufacturing process poses a significant limitation to clinical translation. The use of foetal bovine serum (FBS), while providing nutrients and growth factors, carries the risk of inter-species contamination, including prion, zoonotic, and viral transmission, and may elicit potential immunological reactions due to xenogeneic serum antigens [52]. Moreover, the ill-defined composition of FBS introduces batch-to-batch variation, frequently influencing experimental results [43,53]. 

With MSCs gaining traction in clinical applications, there is a growing demand for large-scale expansion protocols with Good Manufacturing Practice (GMP) standards. Presently, clinical studies have successfully utilised MSCs expanded in media containing FBS without significant adverse effects. However, instances of immunological reactions and anti-FBS antibody formation have raised concerns about potential impacts on therapeutic outcomes. Consequently, there has been a surge in research focusing on replacing FBS with alternatives for expanding MSCs. These substitutes include human serum (HS), human platelet derivatives (HPDs) like platelet lysate (PL) or platelet releasate (PR), and chemically defined serum-free media [54]. HS has shown promise in efficiently expanding ASCs under xeno-free conditions, preserving their phenotypic traits and multilineage potential in a controlled and reproducible manner [55] Additionally, HPL has emerged as a viable substitute for FBS in hMSCs culture, facilitating the adhesion, survival, and proliferation of hMSCs, comparably to FBS [56]. However, the use of human-derived supplements remains controversial due to concerns regarding availability and the potential for disease transmission between donors and recipients. Thus, there is a growing interest in chemically defined serum-free media (SFM) for the production of therapeutic cells intended for clinical applications. 

**Figure 2:**
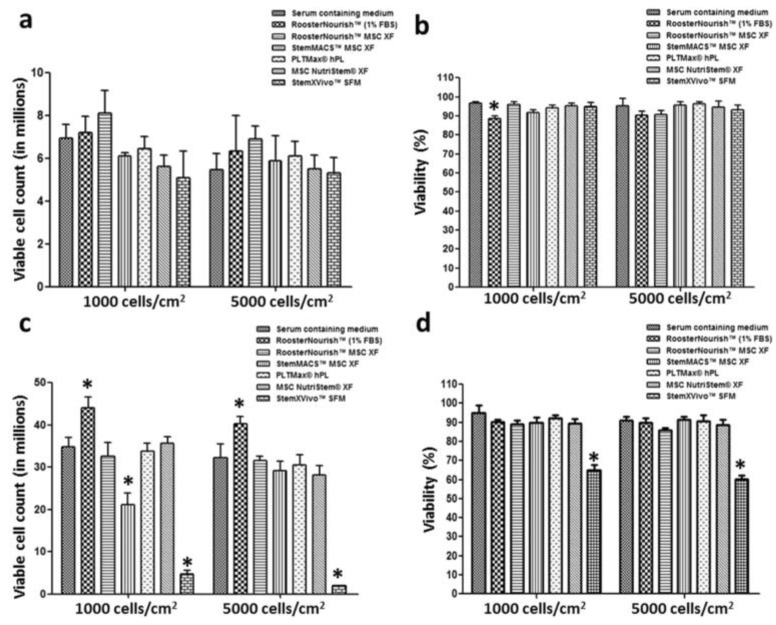
Low-serum/serum-free media supported BM-MSC growth and viability. Comparison of the yield (**a**,**c**) and viability (**b**,**d**) of BM-MSCs expanded in six different commercially available low-serum/serum-free media at P4 (**a**,**b)** and P5 (**c**,**d**) (* *p* < 0.05), Figures are reprinted from Reference [57], with permission from the authors.

Investigations into the differentiation potential, gene expression levels, and viability of MSCs in SFM conditions aim to enhance the understanding of the behaviour and mechanism underlying MSCs in these media [52,57,58,59,60,61]. Notably, studies have shown that ASCs expanded in SFM exhibit lower cellular senescence, reduced immunogenicity, higher genetic stability, and superior osteogenic capabilities compared to cells expanded in FBS-containing media [62]. Cells expanded in SFM maintained their phenotype, with low levels (<5%) of MSC-negative markers (CD34, CD45, and HLA-DR) and high levels (>90%) of MSC markers (CD73, CD90, and CD105). A study assessing the expansion of BM-MSCs in various commercially available low-serum/serum-free media (including RoosterNourish^TM^ (1% FBS), RoosterNourish^TM^ MSC XF, StemMACS^TM^ MSC XF, PLTMax^®^ hPL, and MSC NutriStem^®^ XF) demonstrated that the above growth media supported BM-MSC growth even at low seeding densities, with no notable differences in MSC-specific markers observed between the different media (Figure 2) [57]. Therefore, the use of serum-free media seems to be the direction that will be widely adopted moving forward in BTE, easing the clinical translation of the use of stem cells.

#### 2.1.3. Cell Delivery into Scaffolds

Optimal cell delivery into scaffold constructs plays a vital role in the success of functional bone tissue regeneration. Factors such as a low cell seeding efficiency, inhomogeneity [63], and poor cellular attachment can potentially influence the biological performance of the scaffold [22,64]. The initial seeding density can affect the overall expression of osteogenic genes in the 3D construct due to paracrine signalling between cells. At low cell densities, cellular contact is minimised, compromising bone formation, whereas at extremely high cell densities, cells may be overcrowded, resulting in limited nutrient transport and waste removal, thereby compromising cellular behaviour [65,66]. Hence, achieving optimal cell seeding efficiency with sophisticated cell seeding techniques and strategies is crucial. 

Cells are typically resuspended in expansion media and seeded into 3D-printed scaffolds via static or dynamic cell cultures. A static culture generally involves the dropwise seeding of cells suspended in media onto the scaffold, followed by incubation at 37 °C for a period to allow the cells to adhere to the scaffold [67,68]. Static cultures have drawbacks, such as inhomogeneous cell distribution favouring the periphery of the scaffold, owing to a low diffusion of oxygen and nutrients, and poor waste removal at the scaffold core [68]. Dynamic cell seeding in a bioreactor has demonstrated higher efficiency and improved cell distribution [65], viability, proliferation, and attachment [69] within the scaffold compared to static cell seeding. Birru et al. [70] showed the improved osteogenic differentiation of umbilical cord blood MSCs in a dynamic bioreactor compared to a static culture. 

Bioreactor systems increase mass transport within the 3D construct and allow for the monitoring of controlled environmental conditions, such as the pH, temperature, oxygen concentrations, and nutrient supply. Three classes of bioreactor systems have been frequently used in BTE [65]. Spinner flasks comprise a cylindrical flask with side arms for media exchange and incorporate a stirring element to ensure the convective flow and circulation of media surrounding the 3D construct. The rotating wall vessel comprises two cylinders, outer and inner, with the outer chamber filled with media and the inner chamber containing the 3D construct. The culture chamber then rotates around the horizontal axis to ensure that the culture media surrounding the 3D construct are well circulated. Perfusion systems are the most complex, as they perfuse media directly into the 3D construct using a pump system to provide a more consistent mixing of the media. Different bioreactors can stimulate different outcomes in terms of cellular behaviour and gene expression [71]. Tsai et al. compared the cell viability, proliferation, and differentiation of human BM-MSCs seeded on fibre disks in a static culture, spinner flasks, and a bidirectional-flow bioreactor. The spinner flask enhanced cell viability during the initial two weeks, whereas the bidirectional-flow bioreactor promoted cell proliferation over a four-week duration. Furthermore, the static cell culture demonstrated faster mineralisation after one week, showing higher alkaline phosphatase (ALP) activity, compared to that of the spinner flask and bidirectional-flow bioreactor [72]. 

**Figure 3:**
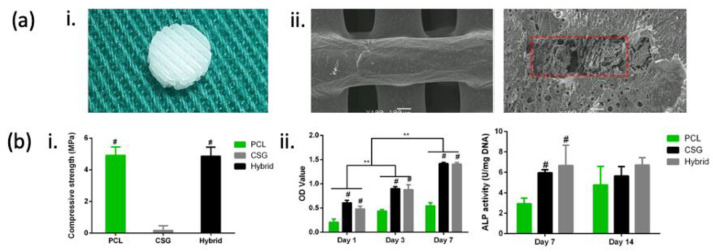
Rabbit BM-MSCs and bone morphogenetic protein-2 (BMP-2) encapsulated in a chitosan hydrogel in a 3D-printed poly(ε-caprolactone) (PCL) scaffold. (**a**) (**i**) Appearance of a 3D PCL scaffold. (**ii**) SEM images of a PCL scaffold (**left**) and hybrid scaffold (**right**). Red rectangle shows the pores of PCL scaffold filled with chitosan gel. Scale bars = 100 μm. (**b**) (**i**) A hybrid scaffold of rBM-MSCs encapsulated in a chitosan hydrogel offers similar compressive strength to a PCL scaffold. (**ii**) CCK-8 assay (**left**) showed that rBM-MSCs remained viable in a hybrid scaffold with the highest ALP activity (**right**) (*^,#^ *p* < 0.05, ** *p* < 0.01). Figures are reprinted from Reference [75], with permission from the authors.

An advantageous method for seeding cells into scaffolds is using hydrogels. Hydrogels for cell cultures and seeding have emerged as a promising option, as they mimic the natural extracellular matrix (ECM) environment, supporting cell adhesion, proliferation, and differentiation [73]. Hydrogels possess high hydrophilicity and adsorption capacity, making them highly beneficial for seeding hydrophobic scaffolds [74]. Various hydrogels have been adopted for cell encapsulation and scaffold seeding. For example, rabbit BM-MSCs and bone morphogenetic protein-2 (BMP-2) encapsulated in a chitosan hydrogel were used to improve the cell seeding efficiency in a 3D-printed poly(ε-caprolactone) (PCL) scaffold. The incorporation of a hydrogel for cell seeding resulted in greater cell retention and proliferation (Figure 3) [75]. These hydrogels can be used in the direct biofabrication of cells onto scaffolds. The biofabrication of various cell types (MSCs, osteoblasts, and ECs), bioactive molecules, or microchannels directly onto BTE scaffolds using 3D-printing techniques has become established in the past few years [76,77,78,79]. However, bioink alone may not be sufficient, in terms of mechanical properties, to support large bone defects. Hence, bioinks in combination with weight-bearing scaffolds should be employed to enable bone regeneration in large bone defects. However, sustaining cell viability, bioactivity, and interactions throughout the 3D bioprinting process and post-printing can be challenging [80]. Numerous reviews of hydrogels for encapsulating cells and growth factors for bone regeneration have emerged in recent years; therefore, these aspects will not be discussed in depth [74,81,82,83].

Low pressure (vacuum) has been used to improve cell seeding efficiency into scaffolds. The vacuum removes air bubbles throughout the scaffold, allowing more cells to penetrate the pores. Torigoe et al. [84] demonstrated a modified low-pressure method for improving cell seeding efficiency and bone formation in a porous beta-tricalcium phosphate (β-TCP) scaffold. It was reported that low pressure did not have a negative effect on the proliferative and osteogenic capabilities of the cells. However, detailed studies on low-pressure-assisted cell seeding are limited. 

Despite advancements in stem cell delivery for BTE, cell distribution and survival in the scaffold after implantation continue to be a concern [17,85]. The function of stem cells, especially in critical-sized bone defects, is impaired due to the poor viability of transplanted stem cells in a large-scale construct. This is because of the less than 200 μm diffusion limit of oxygen and nutrients in a large 3D construct, resulting in unfavourable conditions for cells located beyond this distance [85]. Therefore, the major obstacle to successful bone tissue engineering in large bone defects is vascularisation to improve oxygen and nutrient transport.

### 2.2. Scaffolds

To address the regeneration of large segmental bone defects, it is often necessary and advantageous to combine different aspects of BTE, such as cells, scaffolds, and growth factors, to achieve quick and efficient osteogenesis [86,87,88]. The scaffold serves multiple functions: interconnected pores for cell–cell communication, stiffness for maintaining mechanical stability, acting as a reservoir and adhesive substrate for cells, a filler for the defect space, and a template for bone regeneration. Scaffolds in BTE have evolved to enhance regenerative capacity, driven by an increased understanding of key requirements such as the internal architecture, underlying mechanical properties, chemical composition, and biological features [89]. Recent focus has shifted to the scaffold microstructure and cell-biomaterial interactions [90], recognising that manipulating the macro- and microscale characteristics of the scaffold influences cell adhesion, proliferation, migration, and osteogenic potential, which are crucial for successful bone regeneration [91,92].

The biomaterial should (1) be biocompatible, (2) support cell adhesion and proliferation, (3) have optimal interconnected porosity to allow for vascularisation, (4) possess appropriate mechanical properties, such as the pore size, stiffness, and biodegradation rate in vivo, and (5) be easily fabricated. However, as foreign substances, these biomaterials can provoke adverse immune reactions, leading to excessive inflammation, hindered healing, fibrotic encapsulation, tissue damage, and device rejection. The host immune response can be influenced by factors such as the type of biomaterial (natural or synthetic) and its properties, such as surface chemistry, topography, and bulk properties [93,94]. Hence, understanding the interaction between the host immune system and biomaterials is crucial. Natural and synthetic polymers, as well as ceramics, are commonly used in bone regeneration. Natural polymers mimic molecules found in the body, making them biocompatible with a minimal immune response. However, they may lack the mechanical strength needed for critical-sized bone defects. Synthetic polymers possess good biomechanical and biodegradable properties, but they are more likely to trigger immune responses, although they can be engineered to mitigate this [95]. Ceramics, used in orthopaedic and dental implants, exhibit excellent biocompatibility due to their similarity to native bone tissue in terms of their chemical and structural composition [96]. Researchers are exploring various scaffolds to develop a superior biomimetic product with appropriate properties for promoting osteogenesis and facilitating clinical translation [97]. 

#### 2.2.1. Natural Biomaterials

Natural polymers such as collagen, gelatine, silk fibroin, hyaluronic acid, fibronectin, alginate, and chitosan are biocompatible, biodegradable, and biomimetic, offering optimal cell attachment, growth, and bioactivity. However, disadvantages such as immunogenicity, poor mechanical strength and processability, batch-to-batch variation, and low tunability are associated with natural biomaterials [90]. Nowadays, besides being employed individually for scaffold construction, these materials are used as surface coatings and biological sealants to enhance scaffold properties [98,99,100].

##### Collagen

Collagen, as a major component of the ECM in bone tissues, plays a crucial role in bone tissue engineering. Its use in scaffolds is widespread due to its biomimetic properties and ability to support cell adhesion, growth, and differentiation [101]. The functionality of stem cells is highly dependent on integrin involvement through cell–cell or cell–extracellular matrix (ECM) interactions; thus, it is important to understand the mechanism of integrin binding and cell interaction in the biomaterials. The interaction between cells and collagen is mediated by integrins through specific peptide motifs within the collagen fibres, such as arginine-glycine-aspartic acid (RGD) or Asp-Gly-Glu-Ala (DGEA). This interaction not only facilitates cell adhesion and growth but also influences cell behaviour and fate [102]. 

Collagen for BTE scaffolds can be obtained through direct extraction from animal tissues and subsequent purification or biotechnological production. Collagen hydrogels can be prepared using thermal or chemical crosslinking methods [103]. Collagen type 1, which is highly abundant [104] and accounts for up to 90% of total collagen in bone tissues [98], is a favoured choice for scaffold production, providing a supportive microenvironment for cells. Researchers have explored the combination of collagen with various other scaffold materials to enhance bone regeneration. Coatings such as collagen/chondroitin sulphate (Coll I/CS) on polycaprolactone-co-lactide scaffolds have been shown to promote bone formation in a rabbit calvarial bone defect, compared to a non-coated PCL scaffold [105]. Similarly, the use of a collagen hydrogel to load adipose-derived mesenchymal stem cells (ASCs) into a porous poly(lactic-co-glycolic) acid tricalcium phosphate (PLGA-TCP) scaffold demonstrated homogenous calcified cartilage and bone formation compared to the cell-scaffold construct without the collagen hydrogel [106]. Collagen has also become popular as a bioink for 3D printing [107] due to its abundance, biocompatibility, and mild inflammatory response [102,108]. However, collagen does have limitations, such as low mechanical strength and susceptibility to biodegradation by collagenase. Crosslinking or chemical treatment can address these issues by enhancing mechanical strength while reducing biodegradability. In summary, the unique properties of collagen make it a valuable biomaterial in BTE, and researchers continue to explore ways to optimise its use in scaffolds for improved bone regeneration outcomes. However, owing to the safety concerns associated with animal-derived collagen for clinical applications [108], and as recombinant collagen types are still prohibitively expensive, an alternative material with similar properties remains highly sought-after.

##### Alginate

Alginate is a biocompatible and biodegradable anionic polysaccharide that is inexpensive and well known to support cell growth [99,109,110]. One of its notable features is its ability to undergo reversible gelation via interactions with either of the divalent cations Ca^2+^ and Sr^2+^ [108]. Alginate hydrogels can be prepared using various methods, including ionic interaction, free-radical polymerisation, and click reactions [111]. Using click reactions in gelation provides a controlled and predictable way to engineer the properties of gels, such as their mechanical strength, porosity, and responsiveness to external stimuli. Although alginate-based scaffolds have shown minimal cytotoxicity in vivo [112], they lack intrinsic biological activity. Modifications are often made to incorporate specific binding sites for cell adhesion [99,111]; for example, alginate can be functionalised with RGD peptide, collagen, or gelatine. Studies have demonstrated the effectiveness of alginate-based hydrogels in bone regeneration, particularly when loaded with MSCs. The combination of alginate and MSCs has shown osteogenic and angiogenic properties in various bone defects, including large bone defects [113,114,115,116,117]. Whilst alginate shows promise as a biomaterial in BTE owing to its biosafety and ease of functionalisation, it is associated with challenges such as poor mechanical properties and uncontrolled degradation and presents difficulties in sterilisation and handling. Therefore, further systematic in vivo and clinical translational studies are essential for the successful translation of alginate into clinical settings [118,119].

##### Hyaluronic Acid

Hyaluronic acid (HA) is an anionic non-sulphated glycosaminoglycan (GAG) that is naturally present in the ECM of tissues such as skin, cartilage, and vitreous humour. It is highly hydrophilic and biocompatible, providing a favourable and biomimetic environment for cell adhesion and bone regeneration [108,120]. HA has been used clinically for decades, topically on the skin, and as a vitreous substitute [121,122]. HA is FDA-approved for various products, including dermal fillers (Restylane [123], TriVisc [124], and Triluron [125]), and intradermal injection (SkinVive [126]). One notable advantage of HA is the prospect of cell-free production at a large scale, eliminating the risk posed by animal-derived pathogens [108]. 

Hyaluronic acid has traditionally been sourced from the human umbilical cord, rooster comb, and bovine synovial fluid. However, this extraction process is costly and labour-intensive and raises safety, consistency, and ethical concerns. The biotechnological production of HA from microorganisms, especially recombinant HA from *Streptococcus Zooepidemicus*, has become a preferred method due to its in vitro production eliminating batch-to-batch variations and easing regulatory concerns, making it a promising biomaterial for BTE [123,127]. While biotechnological production works well in small-scale fermenters, it faces challenges in large-scale production due to increased media viscosity affecting mixing and oxygen transfer rates and resulting in the polydispersity of HA. The cell-free production of HA (in vitro) has emerged as a promising alternative for mass production, ensuring a monodisperse HA population [128]. While it has been reported that cell-free production can achieve a high molecular weight, there is a drawback of a low total yield. However, an enzymatic process resulting in a high yield at a lower cost, compared to traditional fermentation methods, has recently been reported [129]. 

Its chemical structure, consisting of several alcohol and carboxylic groups, makes it easily amenable to mechanical and chemical modifications, allowing for tuneable properties [130]. Various methods, including thermal, chemical, Schiff-base reaction, Michael-type addition, or free radical crosslinking, can be employed to prepare HA-based biomaterials [103]. Research has shown that HA plays a regulatory role in cellular processes such as differentiation, migration, angiogenesis, and inflammatory responses [131]. When incorporated into scaffolds, such as PCL, HA coatings have been shown to enhance initial cell attachment, migration/distribution, and proliferation [132] and have demonstrated higher cell seeding efficiency and differentiation, along with a more homogeneous cell distribution relative to non-coated PCL scaffolds [133,134]. 

Native HA has weak mechanical properties, is highly susceptible to in vivo degradation, and is considered unstable. To address these limitations and optimise its utility in BTE, HA modification has been extensively researched [135,136,137,138]. One approach involves the modification of HA with methacrylate groups, allowing for tuneable mechanical strength and degradation rates, making it more suitable for BTE applications [139]. A study by Poldervaart et al. [140] revealed that methacrylated HA (MeHA) possesses intrinsic osteogenicity, exhibiting excellent viability and the osteogenic differentiation of cells. Thus, methacrylated hyaluronic acid (MeHA) is a potential hydrogel for BTE due to its photocrosslinkable and tunable properties. 

With the widespread use of HA in the medical and cosmetic fields, there is growing interest in its potential use in BTE. A critical consideration of employing HA for critical-sized bone defects is its inherent weakness in mechanical strength and rapid degradation rate [137]. Nevertheless, further research is required to optimise the biotechnological and synthetic production of HA and to comprehend its biological functions in bone regeneration [123,128].

##### Fibrin Glue

Fibrin, composed of fibrinogen and thrombin, is a promising biomimetic material for BTE. The interaction between fibrinogen and thrombin results in the polymerisation of these components, forming long strands of fibrin. Fibrin is biodegradable and possesses angiogenic properties, making it well suited for BTE applications [108]. Fibrin has been extensively utilised as a biological sealant, as it is biocompatible, non-cytotoxic, and naturally biodegraded and creates stable haemostasis [141,142]. Additionally, in vitro studies have demonstrated that fibrin glue is a favourable microenvironment promoting cell viability, adhesion, proliferation, and differentiation [143,144,145]. Fibrin has been extensively used for various purposes in BTE, including cell attachment [146], cell delivery [143,147], and bioactive agent delivery [147,148,149]. In contrast to hyaluronic acid, which can be produced by biotechnological methods (see above), fibrin glue is derived from either autologous or xenogeneic sources [150]. Autologous fibrin is obtained from a patient’s platelet-rich plasma (PRP), providing a personalised and biocompatible material [151]. However, variations in PRP quality due to factors such as gender, age, health, and a variety of preparation methods can introduce inconsistencies, limiting fibrin’s potential to become a standardised adhesive agent for BTE [152]. Fibrin derived from animal sources poses the risk of xenogeneic disease transmission, and achieving the optimal concentration of both fibrinogen and thrombin components can be challenging. These factors contribute to the limitations and challenges associated with the use of fibrin in BTE, owing to the need for standardised materials and methods in clinical applications [153]. Moreover, the preparation of fibrin glue and the time taken for clot formation lengthen the medical procedure [154], while instability and variable solubility over time may also impact its acceptance in clinical settings [155] 

Other natural biomaterials including chitosan, gelatine, chondroitin sulphate, and silk have been recently reviewed elsewhere and will not be discussed here [119,156,157,158]. 

#### 2.2.2. Synthetic Biomaterials

Synthetic materials are extensively used in BTE due to their superior mechanical strength, consistent composition, tunability of chemical and physical properties, minimal immunoreactivity, large-scale production capabilities with negligible batch-to-batch variation, and suitability for various biomedical applications. Synthetic materials may provide the advantage of customisation to specific requirements, including the size, shape, mechanical properties, and degradation kinetics [90]. Synthetic materials can be broadly categorised into inorganic materials (e.g., hydroxyapatite, tricalcium phosphate), polymer materials (e.g., polylactic acid, polycaprolactone), and composite materials. These materials offer advantages such as high processability, controlled degradation, and better consistency from batch to batch. However, one limitation of synthetic materials is that they often lack the biological functions present in natural ECM-derived polymers. They may exhibit high hydrophobicity coupled with poor cell adhesion, posing challenges for cell seeding, which subsequently impacts the bone regeneration process [134]. Consequently, modifications are often required to enable tissue engineering applications [103]. One notable consideration is the porosity of synthetic hydrogels, which is on a much smaller scale (nano) than that of cells (micro). This difference may pose a limitation for cell migration and solute diffusion; nevertheless, proper material design considerations can help overcome these challenges [134]. Synthetic hydrogels commonly used as microenvironments for cells include poly(ethylene glycol) (PEG), poly(vinyl alcohol) (PVA), and poly(2-hydroxyethyl methacrylate) (PHEMA).

##### Poly(Ethylene) Glycol

PEG, a synthetic hydrophilic hydrogel, stands out as one of the most extensively researched materials. It has received FDA approval for diverse clinical applications, including drug delivery and bone grafts, and is widely employed in both consumer and medical products [159,160,161,162]. PEG’s versatility is enhanced by its ability to be functionalised with reactive groups on both ends of the polymer, leading to the production of PEG derivatives like PEG diacrylate (PEGDA) and PEG dimethacrylate (PEGDMA) which allows for photocrosslinking through radical polymerisation. The modified PEG can be combined with other materials to create hydrogels with enhanced properties for specific applications [163]—for example, a multi-arm PEG crosslinked with hyaluronic acid (HA) for bone defect repair [164]. This hydrogel demonstrated increased ALP expression and calcium deposition by encapsulated MSCs after 3, 7, and 28 days in vitro, whilst in vivo studies in a rat model showed the enhanced healing of cranial bone defects. Although PEG hydrogels have not been applied to treating critical-sized bone defects in a clinical setting, they possess unique properties that can provide structural support for the defect site to facilitate the repair of bone defects through natural healing mechanisms. Presently, there is still a lack of large-scale clinical studies showing the safety and efficacy of PEG in bone regeneration; thus, further studies for understanding the benefits of PEG in this application will be necessary. 

##### Poly(Vinyl Alcohol)

PVA is another synthetic hydrophilic hydrogel explored for bioengineering tissue scaffolds. PVA possesses a porous structure with high strength, creep resistance, and good water retention [165]. It can be formed through chemical or free radical crosslinking and modified with acryloyl chloride or glycidyl methacrylate to form macromers with reactive pendant hydroxy groups [103,163]. Composite hydrogels, such as PVA/pectin, have been demonstrated to enhance the adhesion and proliferation of osteoblasts, leading to upregulated osteogenic differentiation. These composite hydrogels expedite the bone-healing process in vivo in a rat femoral defect [166]. However, the application of PVA hydrogels in critical-sized bone regeneration is limited due to their low mechanical strength, necessitating their use within a composite biomaterial for bone regeneration [167].

##### Polycaprolactone

Polycaprolactone (PCL) has been widely utilised for the fabrication of 3D scaffolds in BTE in recent years. This popularity is attributed to its high availability, biocompatibility, relatively low cost, and production of fewer toxic degradation by-products compared to other polymers [168,169]. Additionally, PCL is FDA-approved [170] and has a low melting point, making it highly adaptable for processing such as in 3D printing, with excellent malleability and potential for chemical modification [171]. Numerous PCL-based scaffolds have been employed as load-bearing structures for treating critical-sized bone defects, such as collagen-polycaprolactone [172], magnesium oxide-polycaprolactone [173], polycaprolactone-tricalcium phosphate [174], borate bioactive glass-polycaprolactone [175], and polylactic acid-polycaprolactone [176]. Clinical investigations involving PCL implants, conducted in 174 consecutive patients over a decade, have demonstrated the biocompatibility of these implants as synthetic polymers that degrade in the body over a few years [177]. Therefore, PCL is of significant interest as a bone scaffold for clinical translation. 

#### 2.2.3. Composite Polymers

**Figure 4:**
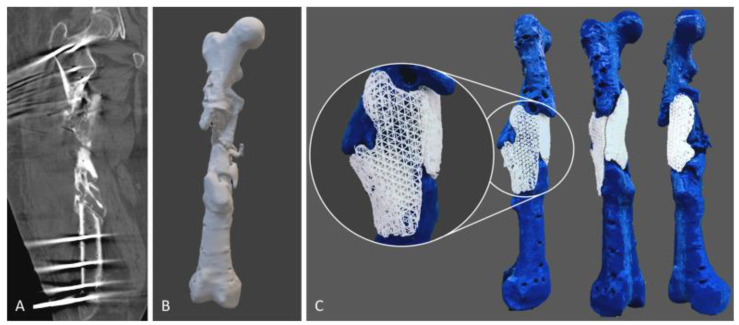
Schematic diagram of the development process of a customised design of a 3D-printed polycaprolactone-tricalcium phosphate (PCL-TCP) scaffold for the patient’s defect. Cross-sectional images of a CT scan (**A**). Based on the CT scan, the surface geometry of a 3D model (**B**) and patient-specific scaffold (**C**) are 3D-printed. The figure is reprinted from Reference [185], with permission from the authors.

Composite materials have emerged as a viable option that offers the benefits of both natural and synthetic materials. Despite possessing great mechanical properties and biocompatibility, synthetic polymers lack inherent bioactivity. Combining synthetic polymers with bioceramics such as β-TCP can enhance the osteoinductivity and osteoconductivity of the resulting scaffold. For instance, composite polymers, such as polycaprolactone-tricalcium phosphate (Figure 4) [178,179,180,181], chitosan-hydroxyapatite [182,183], and collagen-hydroxyapatite [184], are being explored. These materials can be customised to achieve the desired geometry, topography, and surface texture, as well as the preferred biological and mechanical properties [178]. The evolution of scaffolds for critical-sized bone defects has shifted from bioinert materials, such as polymethyl methacrylate (PMMA), polyetheretherketone (PEEK), and ceramics (alumina and zirconia), to biodegradable materials with additional bioactive features to elicit a favourable tissue response in vivo. This includes a range of synthetic (e.g., polycaprolactone [PCL], polylactic acid [PLA], and polyglycolic acid [PGA]) and natural (collagen and hyaluronic acid) polymers, as well as ceramics like calcium phosphates, calcium carbonate, and bioactive glasses.

In recent years, there has been an emphasis on incorporating instructive cues into biomaterials to induce specific biological responses, leading to the development of scaffolds with improved properties and performances in vivo [186]. This approach to scaffold design considers not only the physical characteristics but also the ability to guide and support the biological processes involved in bone regeneration. 

#### 2.2.4. Decellularised Extracellular Matrix (dECM)

Naturally occurring ECM is a biomimetic material that preserves the native tissue environment, promoting cell proliferation and stimulating cell differentiation [187]. It is considered a viable strategy for inducing bone regeneration with low immunological responses and favourable clinical outcomes. dECM can be tissue-derived or produced in vitro through a cell-derived matrix. It can be utilised in various forms, including powder, hydrogel, or electrospun scaffolds [98,188]. The decellularisation process removes cellular components while retaining the ECM components and structure, resulting in a material with a minimal immunological response. However, the processing methods used in tissue-derived dECM production may potentially remove specific components of interest. Despite this, tissue-derived dECM generally preserves the majority of its protein content, bioactivity, and intricate structural framework [101]. In one study, hBM-MSCs seeded in an alginate/solubilised dECM hydrogel, in combination with PLGA microparticles loaded with a growth factor, showed a high induction of collagen deposition and osteoid matrix deposition [189]. In another case, dECM derived from a bovine trabecular bone disc, combined with a patient’s autologous MSCs, was used to treat distal tibia fracture in a clinical setting, resulting in observable bone formation in the graft 6 months post-implantation [190]. Thus, dECM provides a versatile platform to be used in combination with various materials for functional and effective bone regeneration. 

Cell-derived dECM is obtained from a cell culture of autologous cells grown in vitro, and it presents several advantages over tissue-derived dECM. This type of dECM exhibits highly favourable physical and chemical properties and closely resembles the native ECM microenvironment [191]. One advantage of cell-derived dECM is its potential for customisation through the inclusion of various bioactive elements during the culture period [101]. For instance, dECM can be derived from a co-culture of ECs and MSCs, aiming to achieve both angiogenic and osteogenic potential for enhanced bone regeneration [192]. Additionally, the combination of cell-derived dECM with inorganic materials in composite hybrid scaffolds has shown promise in achieving improved mechanical and osteogenic properties [98]. Despite recent advances in the use of dECM for BTE [193,194,195], the field is still in the early stages of development and requires further refinement. One current challenge is optimising the decellularisation process to maximise the removal of cellular components while minimising disruption to the native ECM [187,188]. The majority of decellularisation protocols fail to fully maintain the original 3D structure of the ECM, resulting in alterations to the composition, arrangement, biological activity, and mechanical properties. This poses a significant clinical challenge, emphasising the need for standardised protocols to ensure successful clinical translation. Continued research and advancements in this area hold significant potential for creating tailored and effective biomimetic materials for bone regeneration.

### 2.3. Microenvironment

Despite the increasing use of stem cells in tissue regeneration, challenges such as low cell retention and engraftment and poor long-term cell survival limit the translation of stem cell therapy to general clinical practice. These challenges are often attributed to the inadequacy of the microenvironment in which the stem cells are transplanted. The microenvironment of a scaffold plays a crucial role in providing cells with the necessary substrate for functions such as adhesion, proliferation, differentiation, and vascularisation. It also has a profound impact on determining the fate of cells and the overall outcome of tissue regeneration [99]. Therefore, creating a suitable microenvironment is necessary to provide appropriate cues that support and regulate stem cell function for effective bone regeneration and improve the therapeutic application of stem cells in clinical settings [99].

MSCs engage in interactions with the microenvironment through various protein receptors, including integrins, selectins, and immunoglobulins. These cell–microenvironment interactions have a significant impact on cell adhesion, potentially influencing downstream signalling pathways and the overall biological behaviour of cells. These interactions occur through a combination of biochemical, physical, and mechanical signals [196]. Non-collagenous proteins, such as adhesion proteins (e.g., fibronectin and vitronectin), proteoglycans (e.g., versican, decorin, and hyaluronan), as well as osteocalcin, osteonectin, and osteopontin, play vital roles in ECM construction and the regulation of cell fate. The ECM also plays a role in controlling the delivery of soluble factors, including cytokines (such as growth factors and immunomodulatory factors) and hormones, to regulate cellular behaviour within the microenvironment. Growth factors, such as vascular endothelial growth factor (VEGF), fibroblast growth factor (FGF), platelet-derived growth factor (PDGF), transforming growth factor (TGF), and insulin-like growth factor (IGF), greatly influence cell survival, proliferation, and differentiation [197]. VEGF efficiently stimulates vascularisation, a critical process in tissue regeneration, [198,199], while TGF-β plays a critical role in bone remodelling and the maintenance of bone–tissue homeostasis [200]. Thus, incorporating cytokines into the microenvironment to stimulate bone repair is of particular interest in BTE to harness the signalling capabilities of these factors to enhance the regenerative potential of engineered tissues. 

However, the use of growth factors in tissue engineering also presents challenges such as a short half-life, poor stability in the physiological environment, rapid enzymatic degradation, and high costs [201]. To address these issues, researchers have explored the use of growth factor-derived peptides, also known as oligopeptides, to impart functions associated with the full-length protein. These peptides are short chains of amino acids with a molecular weight typically less than 25 kDa [202]. The advantages of using peptides include full chemical definition, cost-effective synthesis for specific functions, low immunogenicity, and precise conjugation to biomaterials [203,204]. Peptides are recognised as highly selective, therapeutically active, and safe for clinical applications [205]. Oligopeptides, including growth factor-mimetic peptides, have been studied extensively for their ability to regulate various aspects of cellular function such as survival, adhesion, proliferation, and differentiation [204]. To promote cell adhesion in synthetic biomaterials, pro-adhesive peptides can be conjugated into the matrix to enhance cell attachment, proliferation, and differentiation. One example is the tripeptide Arg-Gly-Asp (RGD), a principal integrin-binding domain found in extracellular proteins such as fibronectin and vitronectin. RGD exhibits an affinity for alpha5-beta1 (α5β1) integrin and has been effective in promoting cell binding to various biomaterials, enhancing cell attachment, proliferation, and differentiation [206,207,208,209]. Studies have shown that incorporating RGD into biomaterials such as hyaluronic acid hydrogels can improve cell adhesion and spreading [210]. Additionally, the RGD peptide has been used to promote cell adhesion and distribution in 3D hydrogels [211,212]. Other collagen-mimetic peptides such as the tetrapeptide DGEA [213,214] and GFOGER [214,215] have been employed to enhance the osteogenic potential of MSC grafts. The GFOGER peptide, for instance, was found to enhance vascularisation in critical-sized mice defect models [216]. These peptides offer a promising alternative to growth factors in the clinical translation of BTE.

The microenvironment not only influences cellular behaviour through biochemical signalling but also plays a crucial role in physical and mechanical signalling [197]. Endogenous stress, which includes factors such as the topology and matrix stiffness of the ECM, has been widely reported to regulate stem cell differentiation into various phenotypes. Matrix stiffness, in particular, has been extensively studied in the context of stem cell differentiation [217,218,219]. Stem cells perceive the stiffness of their microenvironment, and this mechanical cue can impact differentiation. For example, a stiff substrate provides a mechanical load that promotes actomyosin assembly and cell spreading, which can influence cell behaviour, including differentiation [218]. The topology of the ECM, or its 3D structure, also regulates the osteogenic and osteoclastic effects in bone tissue. Key regulators of mechanical cues include Yes-associated protein (YAP) and transcriptional coactivator with PDZ-binding motif (TAZ), which are associated with the Hippo pathway. YAP and TAZ can sense the topological and stiffness characteristics of the microenvironment, directly influencing cellular behaviour, including adhesion, morphology, migration, and differentiation [197]. On stiff substrates, the nuclear–cytoplasmic ratio of YAP/TAZ tends to be higher, promoting osteogenic differentiation. Therefore, a stiffer substrate is considered optimal for stimulating MSCs to differentiate into bone. Conversely, on substrates with lower stiffness, the nuclear–cytoplasmic ratio of YAP/TAZ is lower, stimulating MSCs to differentiate into cartilage or adipose tissue [220]. For example, hMSCs were grown on a tunable polyacrylamide hydrogel with a Young’s modulus of 13–16 kPa and 62–68 kPa to investigate how ECM stiffness influences hMSC differentiation. The results showed that hMSCs cultured on the 13–16 kPa ECM exhibited an oval, adipocyte-like appearance, while those cultured on the 62–68 kPa displayed a polygonal, osteoblastic morphology [219]. This also highlights the importance of designing the mechanical properties of a scaffold to guide specific cellular outcomes in BTE. 

Exogenous stress, such as fluid shear stress, also needs to be taken into consideration. Numerous in vitro studies have illustrated that osteoblast cell lines, including MSCs, osteoblasts, and osteocytes, respond to the mechanical stimulus of fluid shear stress, impacting osteogenic differentiation [221,222]. For instance, exogenous and endogenous stress, such as oxidative and replicative stress, can induce senescence in cells [223]. Therefore, creating a microenvironment for successful and efficient bone regeneration and augmentation involves carefully considering both endogenous and exogenous mechanical cues, as well as added biochemical signals.

## 3. Vascularisation

Vascularisation is crucial to the success of bone regeneration, especially in large bone defects. A functional network of blood vessels is essential for providing oxygen and nutrients to the regenerating tissue, enabling efficient osseointegration and supporting the viability of transplanted cells. The lack of sufficient vascularisation in a scaffold can lead to oxygen and nutrient deficiency, the accumulation of waste products, and, ultimately, graft failure [224]. The oxygen diffusion limit of less than 200 μm becomes a critical factor. Beyond this limit, cells may experience hypoxia, leading to cell death and compromising the overall success of the bone construct [225]. 

Different cell types have been incorporated into 3D scaffolds to enhance the vascularisation of large BTE constructs. Most commonly, endothelial cells (ECs) or endothelial progenitor cells (EPCs) are embedded within the scaffold construct to induce vascularisation by forming a capillary-like network. The angiogenic potential of these cells in a 3D scaffold construct has been reported in numerous studies [226,227]. A co-culture with smooth muscle cells (SMCs) or pericytes promotes the stabilisation and functionalisation of the newly formed vessels in the scaffold [228,229]. Additionally, the co-culture of ECs with mesenchymal stem cells is a strategy for promoting both osteogenesis and vascularisation for repairing large segmental bone defects [230,231,232,233]. While in vitro prevascularisation techniques such as optimising the scaffold design and surface topography are useful for constructs on the millimetre scale, translating these successes to constructs on the centimetre scale, especially in a clinical setting, has been challenging. The slow rate of host capillary invasion, with an average sprouting rate of 5 μm per hour (<1 mm in a week), limits the feasibility of current approaches to vascularising large constructs [77]. The vascularisation of large bone defects remains one of the most challenging aspects of successful and functional bone regeneration, hindering the routine clinical translation of BTE as a treatment for critical-sized bone repair [229]. Trauma-induced large bone defects, in particular, face issues of poor vascularisation and impaired healing, making vascularisation strategies imperative for efficient bone regeneration [234]. 

Various successful vascularisation strategies have been adopted in BTE, which can be categorised into cell-based strategies, stimulation by angiogenic growth factors, the biofabrication of vascularised tissue, and even surgery [235]. Despite the progress in these strategies, there is currently no standardised approach for vascularised bone defects. Developing effective and reproducible methods for large-scale vascularisation remains a key area of research in BTE. 

### 3.1. Cell-Based Strategies

The co-culture of osteogenic and vasculogenic cells has emerged as a promising strategy for BTE, aiming to enhance both osteogenesis and vascularisation concurrently. This approach involves the combination of cells such as osteoprogenitors, osteoblasts, and MSCs with vasculogenic cells such as endothelial progenitor cells (EPCs), human umbilical vein endothelial cells (HUVECs), smooth muscle cells (SMCs), pericytes [230,231,232,236,237], and neutrophils [100]. Co-culture can occur with or without direct contact between cell types and can be implemented in 2D or 3D systems. Studies have demonstrated the potential benefits of co-culture in promoting vascular networking and bone formation. The co-culture of MSCs and EPCs on a calcium phosphate scaffold showed improved osteoid tissue formation and neovascularisation in a rabbit large segmental bone defect model compared to acellular and mono-culture scaffolds [230]. 

Another study showed that co-cultured human MSCs and HUVECs on a self-assembled nanomatrix functionalised with a cell-adhesive ligand (RGD) led to higher ALP activity, enhanced osteogenic and angiogenic gene expression, and improved mineralisation compared to a monoculture [238]. While co-culture shows promise, it also introduces complexity, especially in a 3D system, and requires a thorough understanding of the underlying mechanisms and optimal conditions in this biological model. Interactions between different cell types need to be well-characterised to achieve the desired outcomes in tissue-engineered bone. Additionally, the introduction of multiple cell types may pose regulatory challenges that should be carefully considered when planning the target product profile. One notable challenge associated with co-culture is the time required for the formation of a functional microvascular network, which may be in the region of days to weeks, during which period there may be an appreciable susceptibility to ischemia [229]. Controlling the functionality and efficacy of each cell type becomes more challenging due to their complex intercellular communication, emphasising the need for a comprehensive understanding of interactions within the bone scaffold construct. Despite these challenges, co-culture has shown promise as a vascularisation strategy for large-sized bone constructs. 

### 3.2. Angiogenic Growth Factors

The induction of angiogenesis in bone defects often involves the use of angiogenic growth factors, including, but not limited to VEGF, FGF, PDGF, TGF, and angiopoietins (Ang) [224,229,235,239]. These growth factors are commercially available, and incorporation into scaffold constructs or hydrogels is technically straightforward. Growth factors are known to stimulate the migration and proliferation of nearby endothelial cells (ECs), promoting the formation of new blood vessels and establishing a functional vascular network at the defect site. Angiogenic growth factors are often combined with osteogenic growth factors to create a microenvironment that not only supports the differentiation of MSCs into osteogenic lineages but also promotes angiogenesis, leading to the development of a vascular network within the engineered tissue [224,235]. 

VEGF, in particular, is widely known for its ability to enhance neovascularisation in bone scaffold constructs. It not only stimulates ECs but also regulates the release of osteogenic growth factors through paracrine signalling, promoting both angiogenesis and osteogenesis [240,241]. VEGF incorporated into alginate microspheres within a collagen-hydroxyapatite scaffold promoted vascularisation and bone repair in a critical-sized rat calvarial defect model [242]. In another example, a nanocomposite fibrous scaffold loaded with angiogenic growth factors (VEGF and FGF2) in combination with the osteogenic growth factor BMP2 showed that dual growth factor release enhances vascularisation and new bone formation in a critical-sized rat calvarial defect model. Additionally, the loading of multiple growth factors provides a differential release pattern of the various growth factors, with the VEGF diffusion profile sustained for 1 week and BMP2 and FGF2 diffusion sustained over 3 weeks [243]. 

Despite the benefits of angiogenic growth factor delivery in BTE, its therapeutic effects are hindered by several shortcomings, such as a short half-life, rapid diffusion from the delivery site, high costs, and difficulty in controlling temporal and spatial release kinetics and achieving an optimal dosage [201,235]. VEGF has a half-life between 4 h and 24 h. The short half-life limits their effectiveness in clinical settings for large bone defect repair, where a long-term effect is often desired [241]. Moreover, side effects associated with growth factors include oedema, inflammation, ectopic bone growth, immune responses, nerve damage, breathing problems, cancer, and osteoclastic activation. Nevertheless, employing an appropriate delivery system that is selective, nontoxic, and biodegradable holds the potential to significantly enhance the safety and efficacy of growth factor therapies by retaining the growth factor at the bone defect site and restraining the drug from excessive initial dose release [244]. 

### 3.3. Biofabrication of Vascularised Tissue

Advanced techniques such as 3D bioprinting offer a promising avenue for creating vascularised bone tissues with precise control over cell distribution and spatial arrangement. This technique involves the layer-by-layer deposition of bioinks containing cells, growth factors, and biomaterials to fabricate complex 3D structures. The integration of angiogenic cell types, such as endothelial cells, within the bioprinted scaffold aims to promote the formation of a functional vascular network for efficient bone regeneration. Studies have demonstrated the feasibility and efficacy of bioprinting for vascularised BTE. Bioprinting allows for the precise control of the spatial distribution of cells. Various methods of 3D fabrication have also evolved to mimic the native tissue with geometric precision, such as inkjet-, extrusion-based, and laser-assisted printing. Inkjet-based printing dispenses bioink layer-by-layer in a non-contact manner at a high speed and resolution, according to the computer-generated (CAD) digital input. However, the limitations of inkjet systems are that only hydrogels with high gelation properties and low viscosity are suitable for use in these systems. In contrast to inkjet printing, extrusion-based printing expels bioink in a contact-based manner through a microscale nozzle. This is a commonly adopted technique, as it is suitable for materials within a broad range of viscosity, is cost-effective, and is easy to control. However, it is limited by its relatively low printing speed. 

On the other hand, laser-based printing has several attractive features such as compatibility with bioinks of a high viscosity, no nozzle clogging, a high printing resolution and precision, and superior post-printing cell survival [76,235]. However, laser-based printing is relatively expensive and time-consuming [245]. Chen et al. showed that HUVECs bioprinted with MSCs onto a polydopamine-coated calcium silicate (PDACS)/polycaprolactone scaffold exhibited higher expression levels of angiogenic markers, demonstrating the interaction between bone regeneration and angiogenic differentiation [246]. In another study, laser-assisted bioprinting was used to directly print HUVECs into a critical-sized mouse calvarial bone defect filled with collagen, MSCs, and VEGF. This led to organised microvascular structures on the collagen surface, resulting in increased vascularisation and bone regeneration after two months [247]. Nulty et al. [248] demonstrated the bioprinting of a fibrin-based hydrogel comprising both HUVECs and BM-MSCs. The resulting scaffold supported HUVEC sprouting and the establishment of a microvessel network in vitro, while in vivo implantation in a rat femoral bone defect showed enhanced vascularisation and bone formation in a 3D-printed polycaprolactone scaffold, quantified by micro-computed tomography (μCT) angiography. Thus, the biofabrication of vascular networks in a 3D tissue construct is an innovative strategy for in vivo prevascularisation. Together with clinical imaging, a personalised, complex, and biomimetic structure with additives (cells, growth factors, or microchannels) can be bioprinted with specific spatial arrangements for clinical transplantation. 

While bioprinting holds great potential for in vivo prevascularisation, challenges remain for its clinical translation. For clinical applications, patient-specific cells are often required, and presently, there are no standard guidelines and regulations concerning the production of bioink [249,250]. Additionally, different human bones exhibit varying biomechanical properties and microenvironments [80], and the development and standardisation of hydrogel materials, mechanical properties, and cell types for different bone types present challenges. Despite these challenges, bioprinting remains a cutting-edge technology with the potential to revolutionise personalised and biomimetic tissue engineering for clinical transplantation.

### 3.4. Surgical Techniques

One of the major concerns associated with in vitro prevascularisation strategies is the time required for the host to effect the efficient vascularisation of the bone graft. From the time the scaffold is implanted until the bone construct begins to vascularise, cells in the central part of the scaffold may suffer from hypoxia-induced cell death, owing to the oxygen diffusion limit of less than 200 μm. Surgical strategies have been developed to achieve immediate blood supply in the bone implant after implantation. This is essential to ensure cell viability and minimise the risk of hypoxia-related issues during the critical period following implantation [235]. 

**Figure 5:**
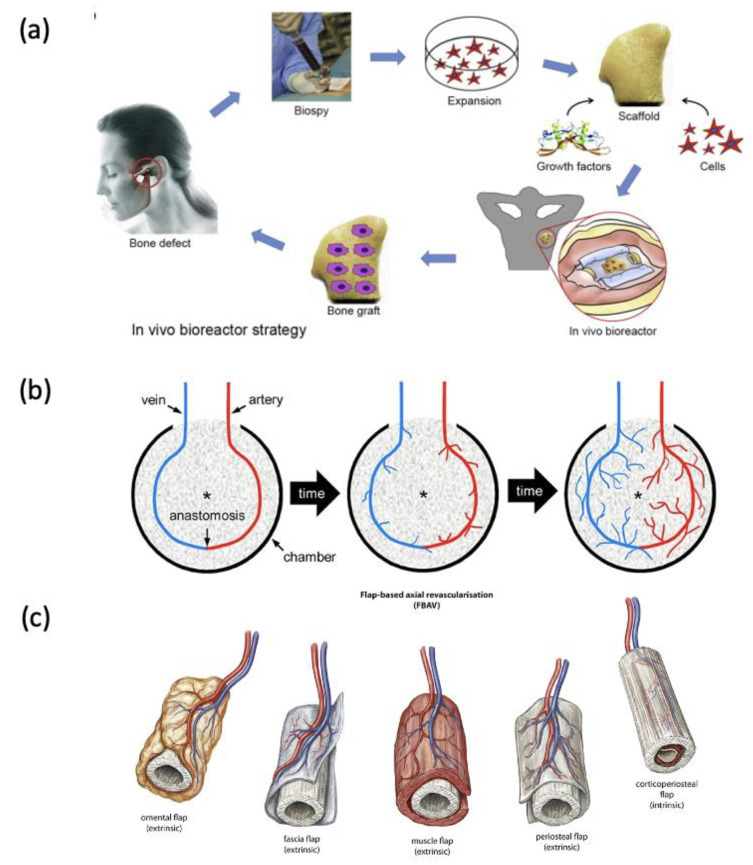
Schematic illustration of (**a**) an in vivo bioreactor strategy, (**b**) AV loop-based vascularisation and (**c**) regenerative matching axial vascularization. Figures are reprinted from (**a**) Reference [252], (**b**) Reference [258], and (**c**) Reference [174], with permission from the authors.

The ‘in vivo bioreactor (IVB)’ technique has been widely used to create vascularised tissues before implantation, offering a promising translational approach by harnessing the patient’s body as a bioreactor to prefabricate new vascularised tissues for reconstructive purposes [224,235,251]. Chambers, corresponding in shape to the defect, are created at a suitable and healthy location of the patient’s body and implanted with the construct. After a period of prelamination, the prefabricated bone graft featuring a flap is transferred to the defect, and the vascular pedicle of the flap is surgically anastomosed to the patient’s vessels, achieving the immediate and complete perfusion of the scaffold construct (Figure 5a) [224,252]. Prefabricated tissue-engineered flaps have been explored in various parts of the human body, such as subcutaneous pocket, periosteal, fascial, muscle, and omental flaps [224,252]. With the IVB approach, leveraging the inherent regenerative capacity of the patient’s cells, vascularisation can be achieved without the use of cells or growth factors, minimising ex vivo cell manipulation while facilitating clinical translation [251,252,253]. The IVB technique has been well established in both preclinical models (pig and sheep) and clinical use cases, with relatively successful outcomes [254,255,256]. Despite its relative success, the IVB technique is complex, requiring a treatment period of several months and necessitating additional surgery, leading to donor-site morbidity [235]. Therefore, the IVB technique can be challenging to translate into clinics as a standardised and routine treatment.

Another surgical technique known as an arteriovenous (AV) loop can be created to generate prevascularised tissues. This strategy involves an AV loop formed by anastomosing an artery with a vein, which is transferred into an enclosed chamber containing a scaffold requiring vascularisation (Figure 5b). The scaffold construct is allowed to vascularise in vivo, by the angiogenic sprouting of new microvessels through the AV loop within the chamber [235,257,258]. The AV technique has demonstrated experimental success in large animal models [259] and clinical studies [260]. However, this technique requires a substantial amount of time for the engineered tissue to fully vascularise, while patients require extended hospitalisation periods, thus limiting its clinical use [235,258]. Both techniques represent innovative approaches to addressing the critical issue of delayed vascularisation in BTE. However, the complexity and lengthy duration of these procedures, along with the associated downfalls, underscore the need for further refinement and optimisation before achieving broader clinical application. Ongoing research aims to enhance the efficiency and practicality of these strategies for more widespread adoption in critical-sized bone regeneration therapies.

Regenerative matching axial vascularisation (RMAV) presents an alternative to current methods that may provide safe, reliable, and predictable outcomes in challenging circumstances [261] (Figure 5c). It has demonstrated success in treating large bone defects in numerous clinical cases [261,262]. The RMAV concept involves using vascularised flaps of regenerative tissue to facilitate tissue regrowth within the scaffold to achieve successful clinical application. For instance, a vascularised corticoperiosteal–cutaneous flap (CPCF) was employed to address a 36 cm bone defect, marking the longest segment of load-bearing bone successfully reconstructed. Radiologically, bone visibility was evident at 9 months, with continuous bone formation observed throughout the scaffold at the 24-month mark [261]. Thus, this technique has been recognised as capable of reconstructing bone defects previously considered unreconstructable, with a reduced risk of implant-related issues and donor-site morbidity [261,262].

Various types of defects in bone tissue require different prevascularisation strategies for successful regeneration. According to the existing literature, co-culturing with ECs appears to be the primary strategy for oral defects [263]. However, as the defect increases in size, such as in the case of large mandibular defects [264] and segmental tibia defects [265], the use of the IVB strategy has been reported to effectively accelerate vascular formation and promote bone regeneration.

## 4. Regulatory Consideration in Bone Tissue Engineering

BTE is a multidisciplinary field encompassing both biological and engineering aspects, and each component presents regulatory challenges. The biological aspects involve the cell source, harvesting, expansion and possibly manipulation for cell seeding onto scaffolds, as well as the incorporation of bioactive factors. The choice of cell source is a pivotal consideration, as it can dictate the regulatory and ethical considerations. For example, the use of human embryonic stem cells (hESCs) raises ethical dilemmas due to the destruction of embryos, sparking debates over the moral status of human embryos [266]. Consequently, alternative cell sources like iPSCs and adult MSCs are often deemed more appropriate, with lesser ethical concerns. However, MSC-based therapies still present some challenges, including the risk of unintended differentiation in vivo and the potential to stimulate tumour growth and metastasis [267]. 

The engineering aspect focuses on creating scaffolds with appropriate biological, mechanical, and structural properties [268]. Such therapeutic products are typically categorized as combination products, thereby following the regulatory guidelines of medical devices. Regulatory issues related to orthopaedic research are extremely complex, and there is no clear regulatory pathway [269,270]. From the initial phase of research, regulatory considerations and concerns should be considered to align the research direction towards future clinical translation. A conducted survey found that research oriented towards regulatory requirements from the beginning has a greater opportunity for clinical approval [270]. 

### 4.1. Regulatory Authority Guidelines

BTE therapeutic products in the USA must meet FDA requirements, meaning each component, material, and biochemical factor involved in the process should also meet FDA requirements [271]. Before a tissue-engineered product is introduced to the pharmaceutical market, it typically undergoes three key stages: product discovery, clinical research, and the preapproval process [272]. In BTE, the type of FDA approval process depends on the constituents of the bone graft and the type of bone graft. 

Generally, bone grafts have four main approval processes, including 510 K clearance, Investigational Device Exemption/Premarket Approval (IDE/PMA), human cells, tissues, and cellular and tissue-based products (HTC/P), and regenerative medicine advanced therapy (RMAT), depending on the type and use of the product. 510 K is a premarket submission for validating that the device is safe, effective, and substantially equivalent to a legally marketed device [273]. An IDE permits the use of an investigational device in a clinical study to gather data on its safety and effectiveness. Subsequently, these data are used to support the Premarket Approval (PMA) [274]. The HCT/P regulation applies to products that consist of human cells, tissues, or cellular or tissue-based products, and the request for RMAT designation must be made with the submission of an existing or new Investigational New Drug (IND) application [275,276]. The RMAT designation is relevant for drugs intended for regenerative medicine therapy, which encompass cell therapy, therapeutic tissue engineering products, human cell and tissue products, or any combination product that incorporates these therapies or products [276].

To facilitate functional bone reconstruction, exploiting the osteogenic potential of MSCs in combination with bioactive factors has emerged as a therapeutic approach for the regeneration of critical-sized bone defects [2,277]. Premarket clinical studies must be performed to demonstrate the safety and efficacy of the combination product. An IDE (21 CFR 812) applies to medical devices, with examples including the Infuse device, OP-1 device, and iFactor device [275,278]. On the other hand, an IND (21 CFR 312) is required for drugs or biologics, and examples include NVD-003 [279] and Aastrom Tissue Repair Cell (TRC) products [280]. NVD-003 is an investigational product intended for critical-sized bone regeneration that incorporates a 3D extracellular matrix along with autologous adipose-derived stem cells to deliver highly specific growth factors and miRNAs aiming to replicate the natural healing process of tissue [279]. The application for IDE/IND must incorporate a description of the product, details regarding the manufacturing process, the design of preclinical studies, and a proposal for the clinical protocol. Hence, the integration of additional components into therapeutic products leads to a more complex regulatory pathway and guidelines. The regulatory frameworks of tissue-engineered and cell therapy products require compliance with the regulations of quality standards such as Good Laboratory Practice (GLP; 21 CFR 58), current Good Manufacturing Practice (cGMP; 21 CFR 820 for medical devices; 21 CFR 210 and 211 for drugs), and current Good Tissue Practice (cGTP; 21 CFR 1271) [272,281]. 

GLP is structured to ensure the integrity of scientific data by maintaining a systematic and accurate record of experimental planning, monitoring, recording, and reporting in non-clinical laboratory studies [282]. The cGMP for medical devices (21 CFR 820) serves as the regulatory framework overseeing the quality system. It governs the facilities and control involved in the design, manufacture, packaging, storage, installation, and servicing of all completed devices intended for human use, ensuring the safety and effectiveness of the final product [281]. cGMP for drugs is divided into the manufacturing, processing, packing, and holding of drugs (21 CFR 210) and, for the finished drug product, includes aspects such as labelling, the production process, equipment management, and personnel (21 CFR 211) [283]. cGTP applies to HCT/P, and its purpose is to ensure an appropriate electronic registration and listing system for establishments engaged in the manufacturing of HCT/P. Additionally, cGTP aims to establish donor-eligibility criteria and implement procedures to prevent the introduction, transmission, and spread of communicable diseases through HCT/P [284]. 

HCT/P is categorised into three main groups: low-risk, middle-risk, and high-risk products. Products falling under both the low- and middle-risk categories are designated as 361 HCT/P, not requiring premarket approval. These include traditional blood and bone marrow progenitor cells and other cell types minimally manipulated for transplantation, intended for autologous and homologous use, which do not involve combinations of cells or tissues. High-risk products fall under the designation of 351 HCT/P. These involve manipulated and gene-modified cell-based products such as cultured cells and tumour vaccines. These high-risk products are used in a manner that is non-autologous and non-homologous, serving a function distinct from their original purpose. In summary, the cGMP regulations, outlined in Parts 210, 211, and 820, apply to HCT/P, contingent on whether the product is classified as a drug, device, or biological product (cGTP; Part 1271). These cGMP regulations act as a supplement to the cGTP requirements [281]. 

Regarding FDA regulations on animal-derived ingredients (ADI), the approach is not straightforward, marked by a clear ‘yes’ or ‘no’. The utilization of ADI requires the implementation of suitable material processing and the purification of animal parts within facilities known as ‘Livestock Processing Establishments’ (LPEs). Stringent controls must be in place to prevent contamination, and additional considerations need to be addressed with the FDA. This includes preventing contamination of the ADI, managing manufacturing contamination risks associated with various pathogenic agents, employing methods for minimizing the pathogenic agent contamination of ADI, and implementing control measures for processes, facilities, and equipment to minimize such contamination [285,286]. Thus, the utilization of ADI entails a regulatory approval process that is more complex and demands thorough justification.

In Europe, the European Medicines Agency (EMA) serves as the regulatory authority for the European Union, comparable to the FDA in the United States. Tissue-based products (TBP) fall under the category of Advanced Therapy Medicinal Products (ATMPs) and are regulated according to European Commission (EC) No. 1394/2007. A TBP combined with a medical device is termed a Combined Advanced Therapy Medicinal Product (CATMP). Within the EU regulatory framework, tissue-engineered products and CATMPs have to undergo the Marketing Authorization Application (MAA), presented to the Committee for Medicinal Products for Human Use (CHMP) in EMA, before they can be released to the market [272]. Presently, the regulatory processes of the US and EU are not harmonised. The consensus is that the FDA approval process is generally slower, more risk-averse, and more expensive compared to the EU. This divergence has the potential to limit access to effective TBP therapies for US citizens compared to Europeans. Moreover, concerns exist in the USA about the reliance on pre-marketing procedures that primarily approve devices based on their resemblance to previously cleared ‘predicate devices’, rather than relying on evidence from clinical trials. Conversely, in the EU, concerns have been raised that the drugs and devices are approved too swiftly, potentially jeopardising patient safety. In recent times, there has been a growing demand to refine approval processes and ensure regulatory uniformity between the USA and the EU [287].

### 4.2. Regulatory Features of Scaffold Materials

Numerous natural materials, including collagen [288,289], hyaluronic acid [289], chondroitin sulphate [289,290], and chitosan [291], have received approval from the FDA for tissue engineering purposes. Collagen is extracted from xenogeneic sources, e.g., bovine and porcine, while glycosaminoglycans (hyaluronic acid, chondroitin sulphate, and chitosan) can be derived from animal sources or produced in bacterial cultures. It is crucial to note that the source of these materials can influence their biocompatibility, functionality, and immunoreactivity. Therefore, the consideration of the product source is vital in selecting natural materials to facilitate clinical translation. Several synthetic polymers are also FDA-approved, such as polycaprolactone (PCL) [292], polyethylene glycol (PEG) [162], and poly(lactic-co-glycolic acid) (PLGA) [293], and have been employed as scaffolds for tissue engineering. Despite lacking the inherent biological function as synthetic polymers, when designed to meet key parameters such as appropriate mechanical properties and hydrophilicity, they can effectively regulate tissue-appropriate responses from cells. The versatility and regulatory approval of these materials make them valuable components in tissue engineering applications. 

The choice of scaffold material significantly influences the regulatory process for a novel tissue engineering strategy. A product deemed ‘substantially equivalent’ to another that is already approved stands a better chance of gaining rapid market acceptance. While striving for improved functionality and innovation is essential, the development of novel materials that surpass existing options may demand substantial efforts to demonstrate the safety and efficacy of the material. This makes the pre-clinical and clinical testing phases both costly and time-consuming [294]. Therefore, it is vital to select a biomaterial that minimises such variability in clinical outcomes. For the reproducibility of experimental and clinical outcomes, the choice of materials for BTE should be made with potential regulatory hurdles and the variability of components in mind. Ideally, a functionalised, synthetic biomaterial with low variability and immunogenicity could facilitate the clinical translation of BTE approaches. 

Regulatory clearance has been one of the biggest hurdles for scaffolds seeking clinical translation, particularly those that are commercially available. Numerous commercial scaffolds encounter difficulties in obtaining regulatory clearance, such as FDA approval in the United States and CE marking in Europe. Table 1 provides a list of these companies offering commercial scaffolds, including reported clinical trials and their status regarding regulatory clearance. 

### 4.3. Regulatory Directives on the Use of Stem Cells for Bone Repair

To facilitate the clinical application of cells, it is crucial to adhere to regulatory requirements. First, cells expanded for large-scale clinical use must be produced following current Good Manufacturing Practice (cGMP) standards [270,300,301]. This ensures the standardisation, reproducibility, and safety of the end product [43]. 

Different cell types are employed in BTE, including adult stem cells, embryonic stem cells (ESCs), and induced pluripotent stem cells (IPSCs) [270]. Adult mesenchymal stem cells can be derived from multiple sources, such as dental pulp, peripheral blood, and, most commonly, bone marrow and adipose tissues [302,303,304]. Despite extensive research and clinical studies with BM-MSCs [305], researchers continue to explore alternative cell sources for bone tissue engineering, including ASCs, IPSCs, and dental pulp stem cells [89,306,307,308,309,310]. One noteworthy example is Nestacell®, a stem cell therapy produced from human immature dental pulp stem cells by the Brazilian company Cellavita. Nestacell® has demonstrated safety in prior clinical trials. It has undergone clinical trials for treating patients with severe COVID-19 pneumonia (NCT04315987), and it is currently in phase three trials for treating Huntington’s disease (NCT06097780).

Despite the longstanding evaluation of different cell types, there are currently no standardised or optimised cell types for BTE. Adult stem cells such as BM-MSCs and ASCs are mostly employed [29] based on their immunomodulatory and anti-inflammatory properties, as well as their ability to promote bone repair due to their inherent multi-lineage potential for differentiation into various bone cell types [29,301] and to support angiogenesis [300]. However, MSCs harvested from adult tissues typically have limited proliferative potential, and obtaining a sufficient number of functional cells for direct transplantation may require extensive in vitro expansion and manipulation before clinical application [301]. BM-MSCs and ASCs are presently considered the preferred source of MSCs, and comparisons between these two cell types are often made [306,311,312,313,314]. In recent years, ASCs have emerged as a favourable source of MSCs compared to BM-MSCs due to their ease of harvest, lesser invasiveness, and high proliferation and differentiation capabilities [315]. The increasing number of studies using ASCs has enhanced our understanding of their physiology and differentiative mechanisms, contributing to the development of novel BTE applications [315]. Nonetheless, ongoing debates between ASCs and BM-MSCs, coupled with the absence of a standardised cell type for BTE applications, continue to drive research across various cell types, hindering clinical translation.

The manufacturing process for producing clinical-grade MSCs according to cGMP guidelines demands great diligence to achieve safe, consistent, and efficient MSCs for regenerative medicine. Key aspects include the MSC donor, cell source, MSC expansion characteristics, culture media, MSC fitness, MSC population enrichment, large-scale culture devices, global-scale MSC production, quantifiable metrics for predicting MSC therapeutic efficacy, and combined advanced therapy medicinal products (ATMP) approach [43]. In regulatory terms, acellular scaffolds will encounter fewer regulatory hurdles compared to composite scaffolds containing a cellular component. The cellular component introduces potential risks such as immunogenicity, tumour formation, issues related to in vitro culture and other manipulations, and concerns about the long-term viability of engrafted cells [294,316]. In vitro cell expansion also raises the risk of culture-acquired genomic abnormality or altered differentiation capacity, posing significant concerns for the clinical use of stem cell products [317]. However, cell-based scaffolds are commonly employed for the repair of critical-sized bone defects, as it is hypothesised that an acellular scaffold may be insufficient to heal large bone defects. 

While various protocols exist for expanding MSCs on a large scale under cGMP conditions, there has been little emphasis on how these protocols affect the critical characteristics and potency of the cells and, consequently, the therapeutic effectiveness [300]. Notably, MSCs used in bone tissue engineering are part of a combination product involving scaffolds, cells, and growth factors, following the regulatory guidelines of medical devices. Adhering to cGMP standards necessitates a fully defined and standardised cell manufacturing protocol, covering processes from cell harvesting and isolation to expansion, differentiation, and transplantation in a clinical setting for the therapeutic use of cells [43]. 

Recent years have seen a rise in in vivo clinical studies [300] and scientific reviews [43,53,301] highlighting the importance of cGMP-grade MSCs as therapeutic agents for clinical use. The scalability of MSC production is a significant concern, as transitioning from the laboratory scale to the clinical grade at a large scale can be both costly and demanding. This process requires standardising the cell source, seeding density, and various components such as the culture medium [53]. However, as manufacturing protocols become more standardised and reproducible, enabling direct comparisons between the efficacy of MSCs across various clinical studies, scalability challenges may be mitigated. 

### 4.4. Regulations on the Usage of Growth Factors for Bone Repair

Growth factors have demonstrated significant potential in regenerating critical-sized bone defects, as evidenced by numerous studies highlighting their effectiveness in enhancing in vitro bone regeneration in such contexts [243,318,319,320]. However, several limitations, including the short duration of action in physiological conditions due to rapid degradation and deactivation, the high cost, unintended side effects such as the promotion of tumour growth, and the risk of systemic toxicity, have impeded their development in effective clinical regenerative treatments. 

Growth factors including FGF, BMP, TGF-β, PDGF, VEGF, and IGF play a role in bone regulation [321]. However, for growth factors incorporated into bone grafts, only BMP-2 (Infuse^®^ Bone Graft) [322], BMP-7 (OP-1) [323], PDGF-BB (Augment^®^ Bone Graft), and P-15 (iFactor Bone Graft) have received FDA approval and been employed in patient treatments [275]. Recombinant human BMP-2 (rhBMP-2) is commercially available as Infuse Bone Graft (Medtronic, Minneapolis, MN, USA) and is combined with an absorbable bovine type 1 collagen sponge, demonstrating significant therapeutic efficacy compared to autologous bone [244]. In contrast, rhBMP-7 has been withdrawn from the market since 2014 [275]. FDA approval is specific to certain growth factors for particular indications. rhBMP-2 is FDA-approved for acute open tibial shaft fractures, alveolar ridge defects, and anterior lumbar spine surgery, while PDGF is approved for ankle and hindfoot fusion [275,324]. Therefore, employing a novel growth factor for a different indication necessitates additional FDA approval. Additionally, the high cost of growth factor therapies has hindered their use in a clinical setting, with studies indicating that the average cost of treatment with BMP-7 was 6.78% higher than that of an autologous bone graft, and BMP-7 itself accounted for 41.1% of the total cost [201,325].

Despite financial and clinical challenges, including side effects associated with FDA-approved bone graft products such as rhBMP-2 (Infuse) and rhBMP-7 (OP-1), these products represent pioneering examples showcasing the effectiveness of integrating biological, biochemical, and pharmacological principles to enhance tissue regeneration. 

In summary, the regulatory pathway for diverse scaffold constructs is contingent on the specific strategy adopted in bone tissue engineering, emphasising that there is no ‘one size fits all’. It varies according to several factors such as the scaffold material, the inclusion or exclusion of cells, the type of cells involved, and the addition of bioactive factors or drugs. Balancing innovation with regulatory compliance is a critical consideration in the field of BTE.

## 5. Current Clinical Landscape and Limitations to Translation

The primary objective of in vitro and in vivo bone tissue engineering studies is the clinical translation of bone tissue constructs to reconstruct bone defects. However, the limited translation of scaffold-based constructs towards clinical applications has been observed, and the field is still in its infancy (Table 2) [326]. Various factors hinder the clinical translation of BTE, including experimental, ethical, and regulatory concerns [326]. Experimental concerns may encompass constraints in supporting the scaffold, limitations in altering scaffold degradation, low attainable cell seeding densities [327], and the variability of components in the scaffold-based bone construct. The multi-disciplinary nature of BTE involving various components results in complex clinical and regulatory requirements, impeding its clinical translation.

To ease clinical translation and overcome regulatory hurdles, it is essential to establish a fully defined protocol with minimised variability. For successful clinical translation, the tissue engineering component must be safe, efficient, cost-effective, and convenient. Concerning cell-based therapies, the clinical translation of bone tissue engineering can be facilitated through different approaches: (1) eliminating the use of cells, (2) minimising the in vitro manipulation of cells, and (3) optimising the choice of cell types. For large segmental bone defects, the incorporation of stem cells into scaffolds has seen significant progress in recent years. Simplifying the in vitro manipulation of cells by eliminating the usage of FBS in culture media, which causes process inconsistency and regulatory complexity, may facilitate the clinical translation of cell-based BTE approaches. Considerations for the choice of cell type include cells that have undergone clinical trials, acceptable cell sources and harvesting procedures, ethical concerns, proliferation rates, and the homogeneity of cells [328].

Over the past decade, academic research on BTE has explored a variety of biomaterials, although only a limited number have progressed to clinical trials (Table 2). Despite the success of certain biomaterials in patient treatment, the field continues to witness the exploration of an increasing number of novel biomaterials, rather than focusing on those that have been investigated, resulting in a significant diversification of research focues and moving away from standardisation. Collagen scaffolds [298,329,330], porous hydroxyapatite (HAp) [331,332], β-TCP [333,334,335], and PCL-TCP [185] have demonstrated the ability to enhance bone healing in critical-sized bone defects, despite presenting complications such as haematoma, inflammation, infection, and dislocation. 

The literature currently describes an extensive list of different scaffold types, each subject to countless modification methods [270]. Each modification aims to enhance the functionality, efficacy, and/or mechanism of the scaffold by adding new components. Currently, there is no gold standard for the type of scaffold to be used, and the choice depends on the properties required for the specific part of the body. For instance, a large tibial segmental bone defect demands a load-bearing scaffold to support the body’s weight. This raises the question of whether researchers should align their investigation of a particular scaffold with a specified application, facilitating the better standardisation of scaffolds for specific bone tissue engineering applications. Furthermore, different cell types require different scaffolds, and it can be challenging to standardise the scaffold type without standardising the cell type [270]. Hence, with the combination of various components, achieving standardisation for clinical translation becomes challenging, particularly if any of the components are non-standardised. This complexity can pose difficulties in clearing the regulatory pathway and progressing to clinical translation.

### 5.1. Challenges of Clinical Translation for Bone Repair Research

#### 5.1.1. Funding for Translational Research

Despite clinical translation being the ultimate objective, the progression of bone tissue engineering beyond academic research has been minimal [327]. This could be attributed to the academic research system, where government research funding organisations place great emphasis on basic research, largely centred on publications and data, with short-term contracts and grants [270]. Academic research tends to emphasise novel, initial discoveries, which are more general, while translational research is usually targeted toward a niche clinical condition, taking into consideration the specific regulatory framework [327]. Although the industry does support research towards clinical translation, translational research is often high-risk, expensive, and time-consuming—especially clinical trials. Therefore, it is difficult for companies to commit to funding translational research, and if they do, there is much less funding for translational research compared to basic research. Translational research also requires collaboration among several multidisciplinary experts and the involvement of clinicians. It is crucial to maintain strong working relationships with stakeholders and engage in consistent discussions for a comprehensive understanding of the relevant clinical needs. However, this can be complicated by the varying needs of different stakeholders [269,270]. 

#### 5.1.2. Regulatory Concerns

Another key factor hindering the clinical translation of BTE is regulatory concerns. As previously described, it is imperative to align the direction of the research with the regulatory requirements from the very beginning. The FDA has outlined the development process for regulatory approval, known as Design Control. This process indicates that the clinical target or condition must be specifically defined before initiating the research [327]. To reduce the risk and increase the chances of FDA approval, ideally, one utilises components that are already FDA-approved or have been used in humans. Trials with preclinical large animal models are necessary before market approval. However, no matter how closely the animal model mimics the human clinical condition, no animal model will perfectly replicate it [269]; the mechanism or efficacy exhibited in the animal model can differ from that of the human model.

#### 5.1.3. Adoption of BTE Strategies in Clinical Settings

To achieve the successful clinical translation of bone tissue engineering, it is crucial to adopt the ‘bedside to bench and back again’ approach. This requires a thorough comprehension of current clinical practices and challenges to identify the existing limitations [281,336]. Even after demonstrating the success and FDA approval of BTE strategies in patients, the implementation of these approaches in clinical settings is not guaranteed, presenting a major barrier to clinical translation. From a clinical perspective, factors such as feasibility, safety, and efficacy, while of the utmost importance, do not guarantee adoption. Other criteria for BTE strategies, including ease of use, minimal manipulation and transportation, cost, and availability, will also significantly influence clinical acceptance [326]. Therefore, researchers should consider these factors when devising their BTE strategy to increase the likelihood of clinical acceptance.

## 6. Future Perspectives on Critical-Sized Bone Tissue Engineering

Experts’ opinions on cell-based bone tissue engineering encompass the following various innovative approaches: (1) Developments in the utilisation of 3D-printed bioresorbable scaffolds, incorporating trace elements of metal like magnesium, which serve to energise cells as scaffold degrades [337,338,339,340,341,342], (2) The exploration of piezoelectric 3D scaffolds, leveraging the effects of electromagnetic fields in BTE [343,344], (3) Progress in the development of physiologic bioreactors aimed at efficiently seeding cells and providing mechanical stimulation [345,346,347,348], (4) The utilisation of induced pluripotent stem cell technologies and investigation into the potential use of exosomes as cell sources [349,350,351,352,353], and (5) The introduction of immune cells, such as neutrophils, to facilitate early vascularisation and auto-regulate vascular growth factors, coupled with surgical techniques [100,354].

Currently, studies are addressing different aspects of critical-sized BTE, such as identifying the ideal scaffold material, determining the type of cells incorporated into the scaffold, defining the optimal microenvironment provided, and developing an effective vascularisation strategy [197]. Despite extensive research, the absence of a standardised procedure for treating large segmental bone defects persists. Presently, inadequate vascularisation is the most significant challenge, which impacts the viability of cell-scaffold constructs and the overall success of BTE in clinical settings. Furthermore, the safety and efficacy of administered cells and exogenous growth factors incorporated into the scaffold remain controversial [224]. 

Key factors hindering the clinical translation of BTE include the lack of collaboration between academic researchers and clinicians, as well as researchers failing to direct their efforts toward clinical translation from the outset. The next-generation BTE is likely to eliminate animal-derived components, which introduce variability in experimental outcomes. Instead, it will employ fully defined, xenogeneic-free components with lower variability and higher chances of FDA approval. 

The major components of BTE, including scaffolds, cells, the microenvironment, and vascularisation for the treatment of large segmental bone defects, require careful consideration for clinical translation. In addition to biological and mechanical property requirements, each aspect involves multiple factors. The scaffold material should not produce a long-term immune response and should be easily reproducible, customisable, and relatively inexpensive. A recent review [355] explored the use of phytogenic materials for bone regeneration in future BTE strategies, highlighting the benefits of cost-effective, highly available, accessible, natural, and biocompatible phytogenic bone grafts. Advancements in 3D-printed bioresorbable scaffolds, incorporating trace elements of metal such as Magnesium [339], Zinc [340], and Silver [341], have been reported to enhance bone regeneration by releasing ions in a sustained manner, thereby promoting cell regeneration [342]. Additionally, harnessing electromagnetic fields with piezoelectric 3D scaffolds has shown significant potential. By leveraging the piezoelectric properties of bone tissue, which generates charges or potentials when stimulated by an electromagnetic field, bone growth can be enhanced [337,338]. Clinical applications of external electrical stimulation and electromagnetic fields have demonstrated efficacy in promoting bone healing [343,344].

In terms of cells, accessibility and abundance are crucial, particularly with a minimally invasive harvesting procedure. Nevertheless, other questions remain.

Could ASCs become the preferred benchmark when compared to BM-MSCs, owing to their minimally invasive isolation procedure and higher abundance? Novel cell types, such as skeletal stem cells (SSCs), have emerged in recent years, with studies suggesting their superiority over MSCs [356,357]. SSCs show potential clinical utility by giving rise to progenitors of bone, cartilage, and stroma, but not fat. Moreover, SSCs from different bone regions (bone marrow and periosteum) play distinct roles in bone maintenance and repair [358]. Bone marrow SSCs contribute to adult steady-state osteogenesis and the mending of smaller, stabilised bone injuries, while periosteal SSCs are responsible for repairing larger, non-stabilised injuries such as fractures. The discoveries in this study raise the prospect of employing a specific cell type to customise the treatment of patients according to the nature of their bone injury. Moreover, it opens the door to identifying new therapeutic targets that could enhance fracture healing. Stem cell-derived exosomes present a promising avenue for bone tissue engineering applications. Unlike stem cells themselves, exosomes derived from stem cells offer several advantages, including non-immunogenicity, easy preservation, the absence of tumorigenic potential and ethical concerns, and exceptional therapeutic potential for numerous diseases [349,350]. Stem cell-derived exosomes inherit similar therapeutic effects as their parent cells, including both embryonic stem cells and adult stem cells [349]. While traditional MSC-based approaches for bone regeneration have limitations such as phenotypic changes during the culture and low cell delivery and survival, there is growing interest in exploring cell-free alternatives using exosomes. These exosomes derived from bone tissue cells have the ability to induce the osteogenic differentiation of MSCs by transferring specific miRNAs to alter target gene expression, thereby stimulating osteoblast proliferation and angiogenesis. Additionally, MSC-exosomes exhibit immunomodulatory effects, contributing to fracture healing by inhibiting the pro-inflammatory factors TNF-α and IL-1β while increasing the anti-inflammatory factor TGF-β [351]. Studies have shown that BM-MSCs-derived exosomes accelerate fracture healing in a mouse model [352] and enhance osteogenesis, angiogenesis, and the bone-healing process in a femoral nonunion rat model [353]. However, there are challenges to overcome, such as the scalability of exosome isolation, the customisation of exosome cargo, understanding their mechanisms of action, and translating these approaches into human clinical trials [351]

Is pre-differentiation of the cells into an osteogenic lineage essential for enhancing osteogenesis in vivo? It is an additional consideration whether to directly implant MSCs or MSCs that have undergone osteogenic pre-differentiation into the scaffold. Presently, pre-differentiation of the cells into an osteogenic lineage is assumed to be essential for enhancing osteogenesis in vitro and in vivo [65,359,360]. However, this remains controversial, with varying outcomes in different preclinical models. Caetano et al. [25] reported that a cellularised scaffold seeded with undifferentiated MSCs seems to be the best strategy for in vivo bone formation in a rat bone defect model, with 15% more bone formation than a scaffold seeded with pre-differentiated cells. On the other hand, Yoon et al. [359] reported that a pre-differentiated ASC cell sheet induced rapid bone healing, while undifferentiated ASCs delayed healing in a canine radial fracture. Pre-differentiated ASCs demonstrated well-organized and mature woven bone, while undifferentiated ASCs demonstrated cartilage formation without bone maturation or ossification at the defect site. Another debatable topic is the optimal pre-differentiation duration for bone regeneration, since brief osteogenic induction may be insufficient for inducing osteogenic differentiation, while a prolonged period of osteogenic differentiation may elicit an apoptotic process [65]. However, from a clinical point of view, a single-step surgical technique for administering freshly isolated cells directly to patients in the operating theatre would be preferred [361].

Another pressing question is, what innovative approaches can be developed to enhance the integration of stem cells into scaffolds for effective bone regeneration in clinical settings? One promising avenue involves the use of innovative techniques, such as photocrosslinkable hydrogels, which enable the incorporation of cells, drugs, and growth factors into the scaffold. For instance, GelMA hydrogels containing hMSCs and HUVECs were employed and loaded into a biodegradable polylactide (PLA) scaffold. Recent results [362,363] revealed the uniform evolution and expansion of cells in the 3D space during the culture period. This approach demonstrated remarkable bone regeneration, highlighting its potential for addressing challenges associated with uniformly loading cells in critical-sized bone defects. In recent years, there has been an increasing interest in utilising physiologic bioreactors to facilitate efficient cell seeding and provide mechanical stimulation [345,346,347,348]. In vivo studies demonstrated the efficacy of neutrophil-mediated bone regeneration, with neutrophil-treated groups showing a higher bone volume fraction in rabbit calvarial defect models [100]. Additionally, a novel co-culture model involving osteoblasts, endothelial cells, and neutrophils revealed that neutrophils significantly enhanced both angiogenesis and osteogenesis within the tissue construct [354].

The microenvironment should be sustainable over a specific period and homogeneous throughout the scaffold. Vascularisation techniques should achieve rapid and homogeneous vascularisation throughout the scaffold. An emerging vascularisation technique for critical-sized bone defects is the introduction of immune cells such as neutrophils to address the need for early vascularisation and auto-regulate vascular growth factors. Immune cells play an imperative role throughout the whole process of bone healing [95,364]. From a clinical perspective, BTE strategies should prioritise convenience and minimal material transportation. Ideally, the entire cell-scaffold construct should be fabricated in a single setting, integrating isolated cells directly and minimising in vitro expansion and manipulation. This approach would allow for prompt implantation, eliminating the need for additional surgery, and extra materials, addressing safety concerns associated with the handling of cells and materials, thereby reducing costs, saving time, and increasing the clinical supply.

Lastly, the progress of interdisciplinary collaborative efforts across major stakeholders, including scientists, clinicians, and engineers, could exponentially accelerate the advancement of BTE strategies in clinical settings.

## 7. Conclusions

In summary, various factors conspire to impede the clinical translation of BTE. The cited literature reveals that this is a growing field with significant commercial potential, with China, USA, and Germany emerging as the top three countries leading research efforts. Among the critical research topics, the vascularisation of bone tissue engineering stands out, necessitating a blend of basic bioengineering science and surgical techniques to achieve success in clinical trials and ultimately save patients. To facilitate this multidisciplinary process, various stakeholders must reorient their views, goals, and direction. This includes researchers aligning their research towards clinical and regulatory requirements, government institutions allocating funding for translational research, and fostering collaboration and communication between clinicians and academic researchers to enhance clinical translation efficiency. If these factors are considered, they will undoubtedly facilitate the clinical translation of BTE, paving the way for standardised and reproducible solutions for critical-sized bone defects.

## Figures and Tables

**Table 1:** Companies with commercialised 3D scaffolds for the treatment of bone defects and their status of regulatory clearance.

Company	Material	Human Clinical Studies	Regulatory Clearance
Osteopore®	PCL-TCP	Four patients (two Tibial, calvarial, and mandibular) [261] Four patients with lower-extremity large bone defects [185] BONE-RECON trial for critical-sized lower limb defect (ongoing) [295]	FDA ISO 13485 FDA (US) CE marking (Europe) KFDA (Korea)
BellaSeno GmbH	PCL	46-year-old male patient with a 14 cm segmental bone defect of radial shaft [296]	ISO 13485
Dimension Inx CMFlex™	Calcium phosphate (CaP) and poly(lactide-co-glycolide) (PLG)	Mandibular angle augmentation and maxillary segmental osteotomy (ongoing) [297]	FDA
Medtronic INFUSE® Bone Graft	Recombinant human bone morphogenetic protein-2/absorbable collagen sponge (rhBMP-2/ACS)	Controlled, randomised study of 450 patients for the treatment of open tibial fractures [298] Observational study of 86 patients for the treatment of tibial fractures [299]	FDA
A.D.A.M Bone Graft	Modified biopolymer and ceramic bio-glass	N.A.	510(k)

**Table 2:** Clinical developments in the field of bone tissue engineering with scaffolds and/or stem cells for large segmental defects (accessed at clinicaltrials.gov on 24 May 2023).

Clinical Trial #	Study Titles	Phase	Treatment	Sponsor/Country	Duration	Status
NCT05693558	NVD-003 in the Treatment of Congenital Pseudarthrosis of the Tibia	1	Condition: Congenital Pseudarthrosis or Tibia NVD-003 is a scaffold-free 3D osteogenic graft derived from autologous adipose stem cells which become embedded in their extracellular matrix and combined with hydroxyapatite/beta-tricalcium phosphate (HA/βTCP) particles.	Novadip Biosciences	24 November 2022–December 2024	Recruiting
NCT05520125	Treatment of Patients with Bone Tissue Defects Using Mesenchymal Stem Cells Enriched by Extracellular Vesicles	1 and 2	Condition: Segmental Fracture—Bone loss Mesenchymal stem cells enriched by extracellular vesicles to treat patients with segmental bone tissue defects	Institute of Biophysics and Cell Engineering of National Academy of Sciences of Belarus	1 November 2022–31 December 2023	Not yet recruiting
NCT05668182	A Case Series: TRUMATCH Graft Cage for Segmental Long Bone Defects	NA	Condition: Tibia, Humerus, Femoral Fracture, and Long Bone Segmental Defect TRUMATCH Graft Cage (3D-printed personalised resorbable implant) implanted into a critical-sized humerus, femur, or tibia segmental defect through surgery	University of California, San Diego	20 June 2021–29 June 2026	Recruiting
NCT03941028	Clinical Effects of Large Segmental Bone Defects with 3D Printed Titanium Implant	NA	Condition: Large Segmental Bone Defect caused by Trauma, Infection, or Tumor Polyporous 3D-printed titanium implant implanted into patients to treat large segmental bone defects	Peking University Third Hospital	12 January 2019–30 December 2021	Unknown
NCT01958502	Evaluation the Treatment of Nonunion of Long Bone Fracture of Lower Extremities (Femur and Tibia) Using Mononuclear Stem Cells from the Iliac Wing Within a 3-D Tissue Engineered Scaffold	2	Condition: Nonunion of Fracture in Lower Extremities Mesenchymal stem cells derived from iliac bone marrow with BMP2 in a collagenic 3D scaffold placed in a nonunion site by a surgical approach	Emdadi Kamyab Hospital, Mashhad, Khorasan, Iran, Islamic Republic of National Taiwan University of Science and Technology	July 2013–November 2014	Unknown
NCT01842477	Evaluation of Efficacy and Safety of Autologous MSCs Combined to Biomaterials to Enhance Bone Healing (OrthoCT1)	1 and 2	Condition: Delayed Union after Fracture of the Humerus, Tibial, or Femur Implantation of autologous cultured mesenchymal stem cells expanded in a GMP facility, mixed with granulated biphasic calcium phosphate	Institut National de la Santé Et de la Recherche Médicale, France	May 2013–5 February 2016	Completed (No results posted)
NCT02609074	Pilot Clinical Trial of CPC/rhBMP-2 Microffolds as Bone Substitute for Bone Regeneration	4	Condition: Bone Fracture CPC/rhBMP-2 micro-scaffolds and CPC paste (control) implanted into patients of tibial plateau fractures, proximal humeral fractures, or calcaneal fractures	East China University of Science and Technology	March 2013–October 2015	Completed (No results posted)
NCT02209311	Effectiveness and Safety of Method of Maxilla Alveolar Process Reconstruction Using Synthetic Tricalcium Phosphate and Autologous MMSCs	1 and 2	Conditions: Partially Edentulous Maxilla, Alveolar Bone Atrophy, and Alveolar Bone Loss Implantation of a tissue-engineered construct containing autologous multipotent MSCs obtained from an oral mucosa biopsy sample and synthetic tricalcium phosphate	Central Clinical Hospital w/Outpatient Health Center of Business Administration for the President of Russian Federation	September 2014–March 2018	Unknown
